# Supplementary material for: Up-to-fivefold reverberating waves through the Earth’s center and distinctly anisotropic innermost inner core
Source: Nat Commun. 2023 Feb 21;14:754. doi: 10.1038/s41467-023-36074-2 (PMC9944935; doi:10.1038/s41467-023-36074-2)

Supporting Information for

Observations of up-to-fivefold reverberating waves through the Earth's center and distinctly anisotropic innermost inner core

Thanh-Son Phạm[1] and Hrvoje Tkalčić[1]

[1]Research School of Earth Sciences, The Australian National University, Canberra ACT, Australia

Supplementary Note

The content of this file is as follows:

In the Supplementary Methods, we present a series of finite-frequency simulations demonstrating the sensitivity of the long-period signals to a 650-km-radius innermost inner core (IMIC). In addition, Figures S1 to S9 support the points from the main text. Figures S10 to S13 are mentioned in the Supplementary Methods.

In the Supplementary Figures, we supplement 32 figures featuring 16 sets of differential travel time measurements for the pairs of PKIKP multiples (similar to Figures 5 and S9).

All supplementary references mentioned in this text are already cited in the main text.

Supplementary Methods

We perform full-waveform experiments to (*i*) benchmark the sensitivity of the exotic reverberating arrivals to the IMIC, (*ii*) demonstrate the theoretical accuracy, and (*iii*) identify limitations of the differential travel time measurements between pairs of exotic PKIKP multiples in probing the anisotropic strength of the IMIC.

Experiment setup

We use the spectral element method in 2D (e.g., Komatitsch et al., 2002) to synthesize waveforms in an Earth's cross-section, described by the ak135 model's elastic properties. In the *specfem2d* package, a built-in mesh generator for an Earth's cross-section is provided as an example (global_Earth_ak135), and we determine mesh sizes to achieve the minimum period of ~7.3 seconds. An isotropic source is located at 200 km depth, and two receiver arrays are equally spaced to span 0–50° and 155–180° epicentral distance ranges. In each range, there are 101 receivers, so the inter-receiver intervals are 0.5° and 0.25°, respectively.

An innermost inner core (IMIC) in the Earth model is introduced by a relative increase of P-wave speed within the centermost 650-km radius from the background model (see a simulation snapshot in Figure S10). We use different isotropic IMICs to simulate the IC probes from different angles with respect to the Earth's rotation axis. Synthetized waveforms, the output of the finite-frequency simulations, are filtered in the same way as the real waveforms, more specifically, bandpass-filtered between 7–13 seconds using a Butterworth filter, two passes, and three corners. In Figures S11 and S12, the waveforms are windowed around the predictions of the PKIKP multiples.

Sensitivity to a 650-km-radius IMIC

Firstly, we examine synthetic waveforms from the original ak135 model (Kennett et al., 1995) to warrant the use of ray-theoretical predictions as a reference for residual travel time measured at 7–13 s (Equation 3 in the main text). Synthetic waveforms of the original ak135 model align well with the ray-theoretical predictions (Figures S11A and S12A). Also, their stacked waveforms of both PKIKP3-PKIKP and PKIKP4-PKIKP2 pairs overlap (Figures S11B and S12B). Thus, we confirm the agreement between finite-frequency and ray-theoretical results.

To prove the sensitivity of the podal and antipodal PKIKP multiples to a 650-km-radius IMIC, we compare the synthetic waveform of the original ak135 model with a model

with the IMIC's P-wave speed increased by 3%. In Figures S11C and S12C, there are gradual discontinuities at ~16° and 32° in the plots corresponding to epicentral distances at which PKIKP and PKIKP2 ray paths graze the IMIC boundary. Such discontinuities for PKIKP3 and PKIKP4 are beyond the observed distance range, but due to the faster IMIC, their stacked waveforms arrive early compared to their PKIKP and PKIKP2 counterparts (see Figures S11D and S12D). This experiment benchmarks the exclusive sensitivity of the PKIKP multiples to the IMIC in the 7–13 s period band.

Measurement robustness and limitation

We qualitatively assess the accuracy of the residual measurements by cross-correlating PKIKP multiple waveforms in several epicentral brackets. The IMIC strength varies from 0% (the original model) to 5% in a series of waveform simulations. Because the IMIC radius is around 50% of the IC, the observed P-wave speed increase averaged for the entire IC (Equation 3 in the main text) is ~50% of the input IMIC strength (see the prediction lines in Figures S13A and S13B). Each observed value is measured by the time shift with respect to prediction using synthetic waveforms stacked in corresponding brackets, which is then converted to relative speed perturbation, $\Delta v/v$, using Equation 4.

As can be seen in Figures S13A and S13B, we have a robust recovery of the input IMIC strength using the PKIKP4-PKIKP2 pair in the podal setting (<50°) and the PKIKP3-PKIKP pair in the antipodal setting (>155°) for almost all epicentral-distance brackets. However, the observed $\Delta v/v$ markedly over-estimate the theoretical prediction in the two epicentral distance brackets of 30–40° and 40–50° for PKIKP4-PKIKP2, which is due to large discrepancy of PKIKP2 and PKIKP4 ray paths (see Figure 1B). The worst-case scenarios apply to two analyzed events, 20100812 and 20120930 (see Figure S13C). However, it is noted that when the actual observed value of $\Delta v/v$ < ~1.5% (see Figure 3A), the observation is sufficiently close to the theoretical prediction (see Figure S13A).

Thus, overall, the sensitivity numerical simulations attest to the robustness of the differential travel time measurements presented in this study.

Supplementary References

Kennett, B. L. N., & Gudmundsson, O. (1996). Ellipticity corrections for seismic phases. *Geophysical Journal International*, *127*(1), 40–48. https://doi.org/10.1111/j.1365-246X.1996.tb01533.x

Kennett, B. L. N., Engdahl, E. R., & Buland, R. (1995). Constraints on seismic velocities in the Earth from traveltimes. *Geophysical Journal International*, *122*(1), 108–124. https://doi.org/10.1111/j.1365-246X.1995.tb03540.x

Komatitsch, D., Ritsema, J., & Tromp, J. (2002). The Spectral-Element Method, Beowulf Computing, and Global Seismology. *Science*, *298*(5599), 1737–1742. https://doi.org/10.1126/science.1076024

Li, C., van der Hilst, R. D., Engdahl, E. R., & Burdick, S. (2008). A new global model for P wave speed variations in Earth's mantle. *Geochemistry, Geophysics, Geosystems*, *9*(5). https://doi.org/10.1029/2007GC001806

Li, X., & Cormier, V. F. (2002). Frequency-dependent seismic attenuation in the inner core, 1. A viscoelastic interpretation. *Journal of Geophysical Research: Solid Earth*, *107*(B12), ESE 13-1-ESE 13-20. https://doi.org/10.1029/2002JB001795

Simmons, N. A., Myers, S. C., Johannesson, G., & Matzel, E. (2012). LLNL-G3Dv3: Global P wave tomography model for improved regional and teleseismic travel time prediction. *Journal of Geophysical Research: Solid Earth*, *117*(B10), B10302. https://doi.org/10.1029/2012JB009525

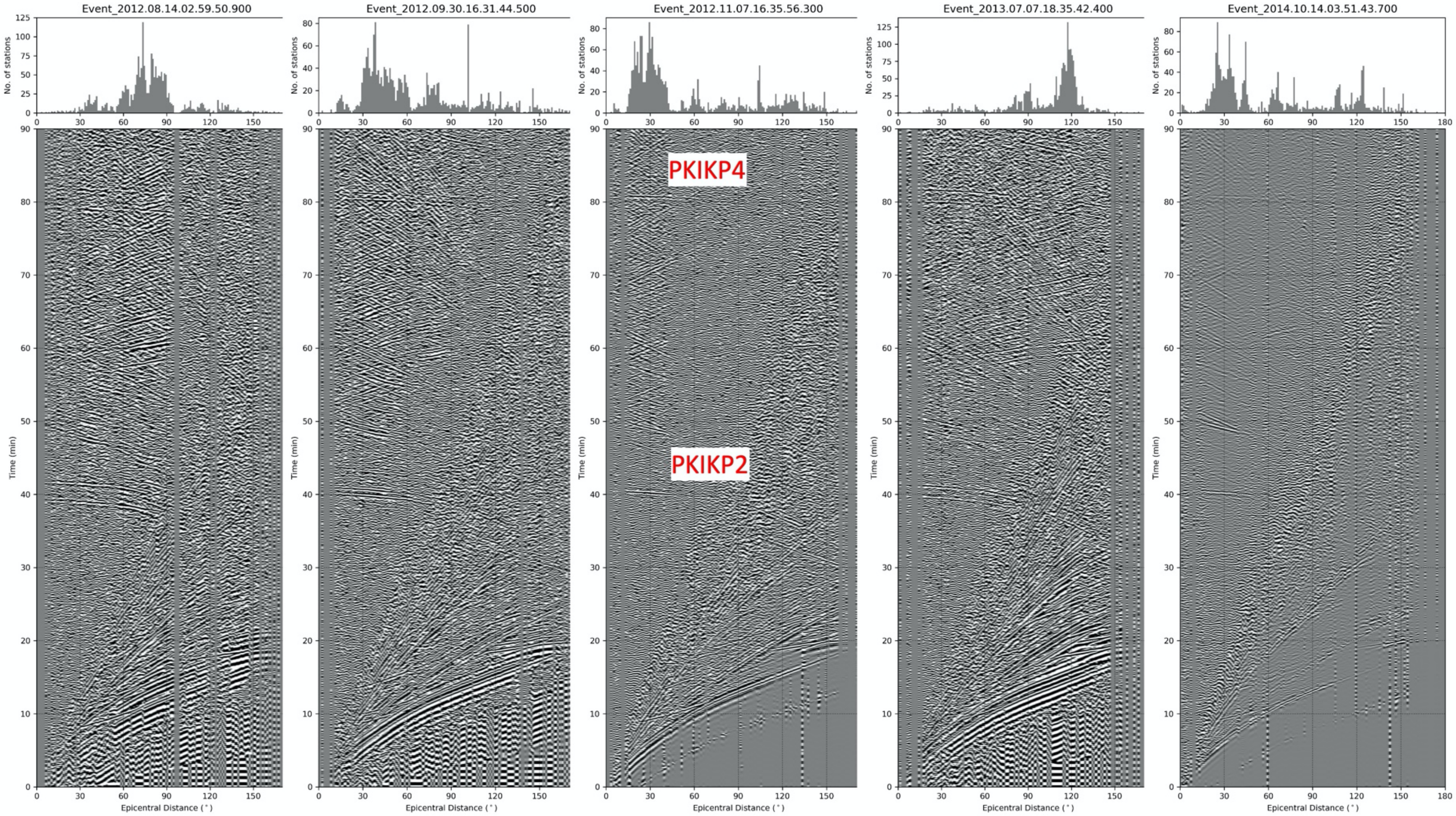

Figure S1. Global stack examples express clear exotic podal and antipodal reverberations. Panel titles note event origin times.

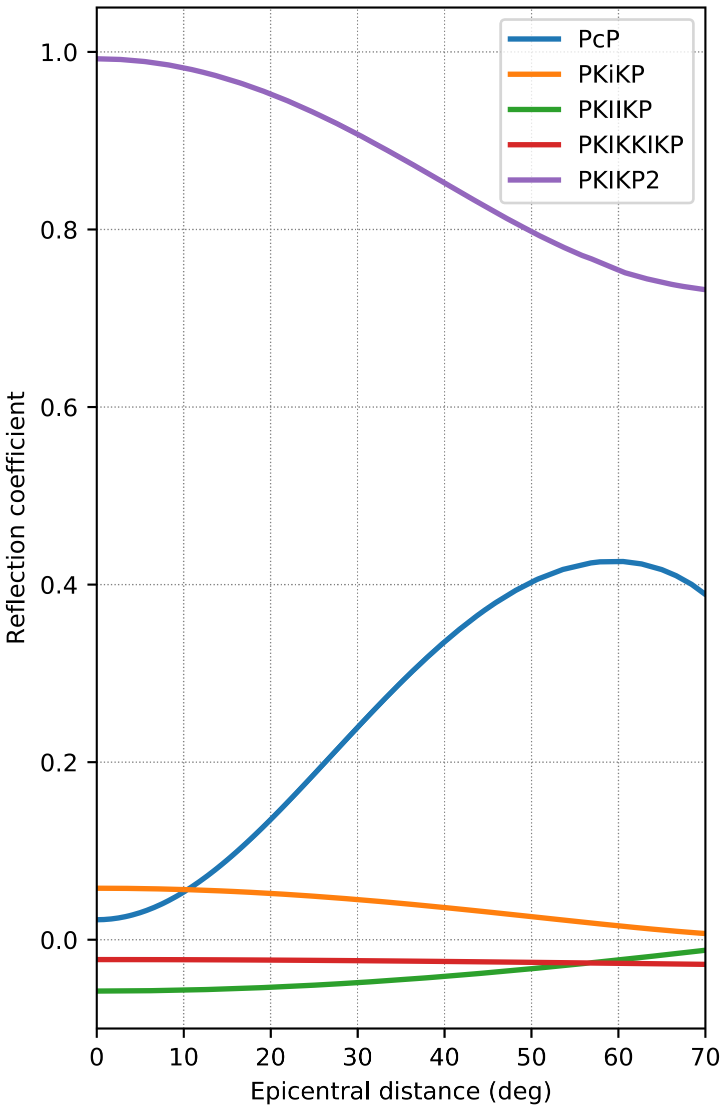

Figure S2. Theoretical reflection coefficients at major Earth's internal interfaces as function of epicentral distances: upper reflection at CMB (PcP), upper reflection at ICB (PKiKP), under-side reflection at ICB (PKIIKP), under-side reflection at the CMB (PKIKKIKP), under-side reflection at the Earth's surface (PKIKPPKIKP).

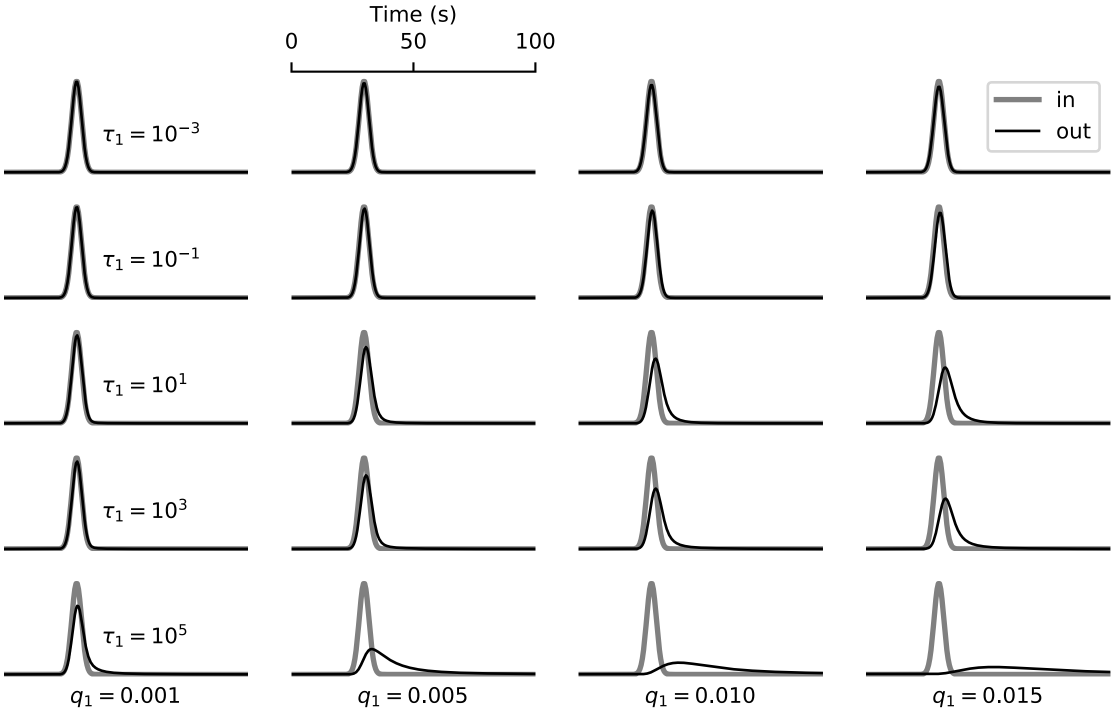

Figure S3. Attenuation operators using the absorption band method (Li & Cormier, 2002) convolved with a given wavelet having 10 s central period. The travel distance is comparable to the diameter of the inner core. Amplitudes are normalized to the incident wavelet. τ_1 represents the lower end of the absorption band, while q_1 is the inverted value of the quality factor Q_1 corresponding to τ_1. (Readers are referred to the original reference for the complete mathematical representation). Unless the extreme value is used for τ_1, the long-period signal (10 s) only weakly attenuates while propagating in the inner core.

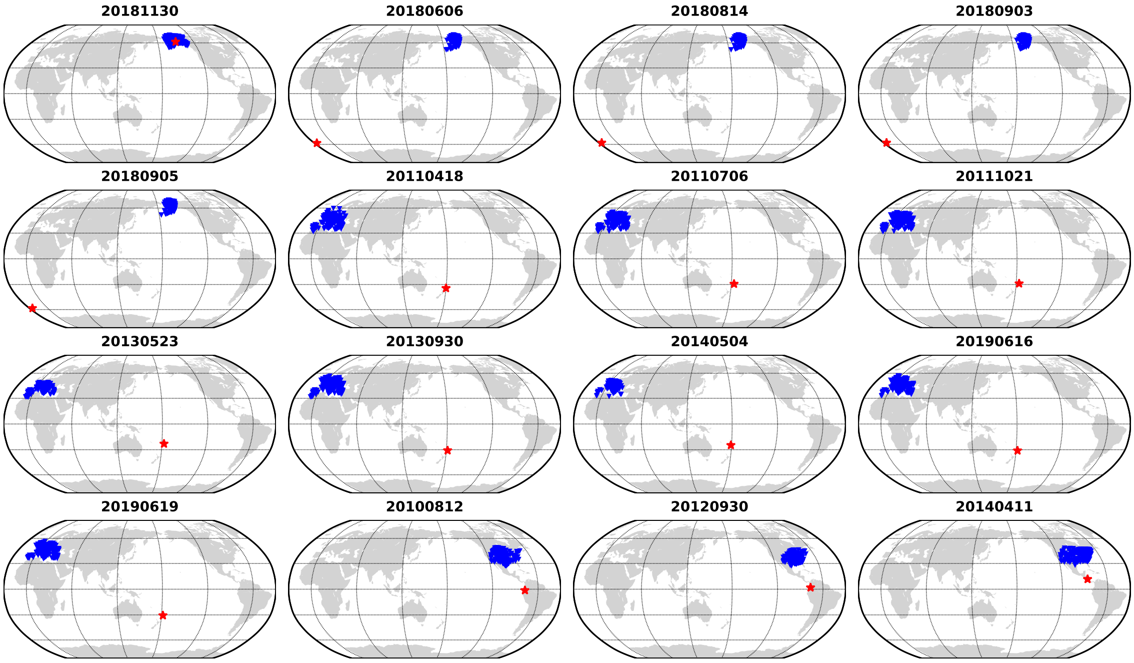

Figure S4. Location maps for 16 high-quality events producing measurements of differential travel times for arrays in the podal and antipodal distance ranges (<50° for PKIKP4-PKIKP2 pairs, and >155° for PKIKP3-PKIKP pairs). Events are denoted by red stars. Seismic stations are shown by blue triangles.

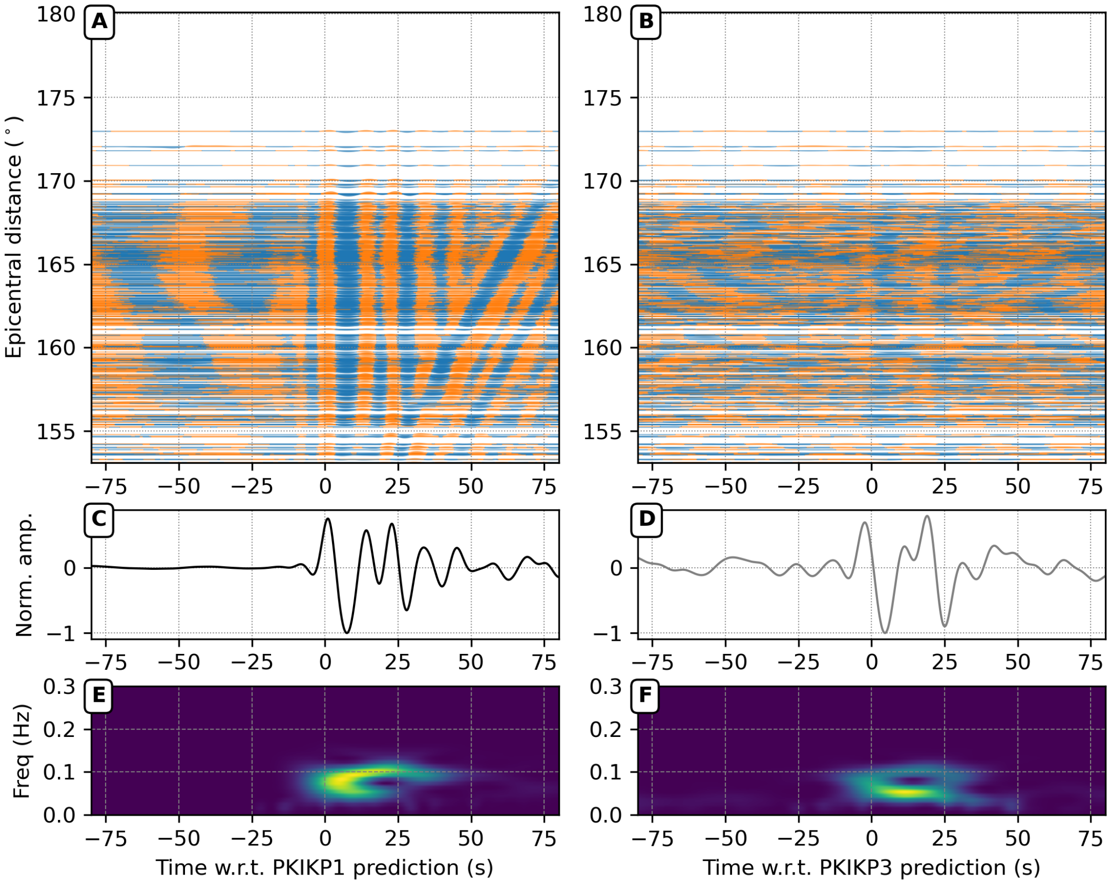

Figure S5. Observations of PKIKP and PKIKP3 phases in the seismic wavefield from Mw 6.9 Tonga region, 19/06/2019. (A) Seismic records from the Alaskan network are aligned predictions of PKIKP1 arrivals, corrected by the Earth's ellipticity (Kennett & Gudmundsson, 1996). The waveforms are bandpass filtered in the period band of 7–13 seconds. (C) Linear stack of individual waveforms. (E) The spectrograms of stacked waveforms before filtering show the frequency content variation as a function of time. (B, D, F) Similar to A), C), E) but for the PKIKP arrivals.

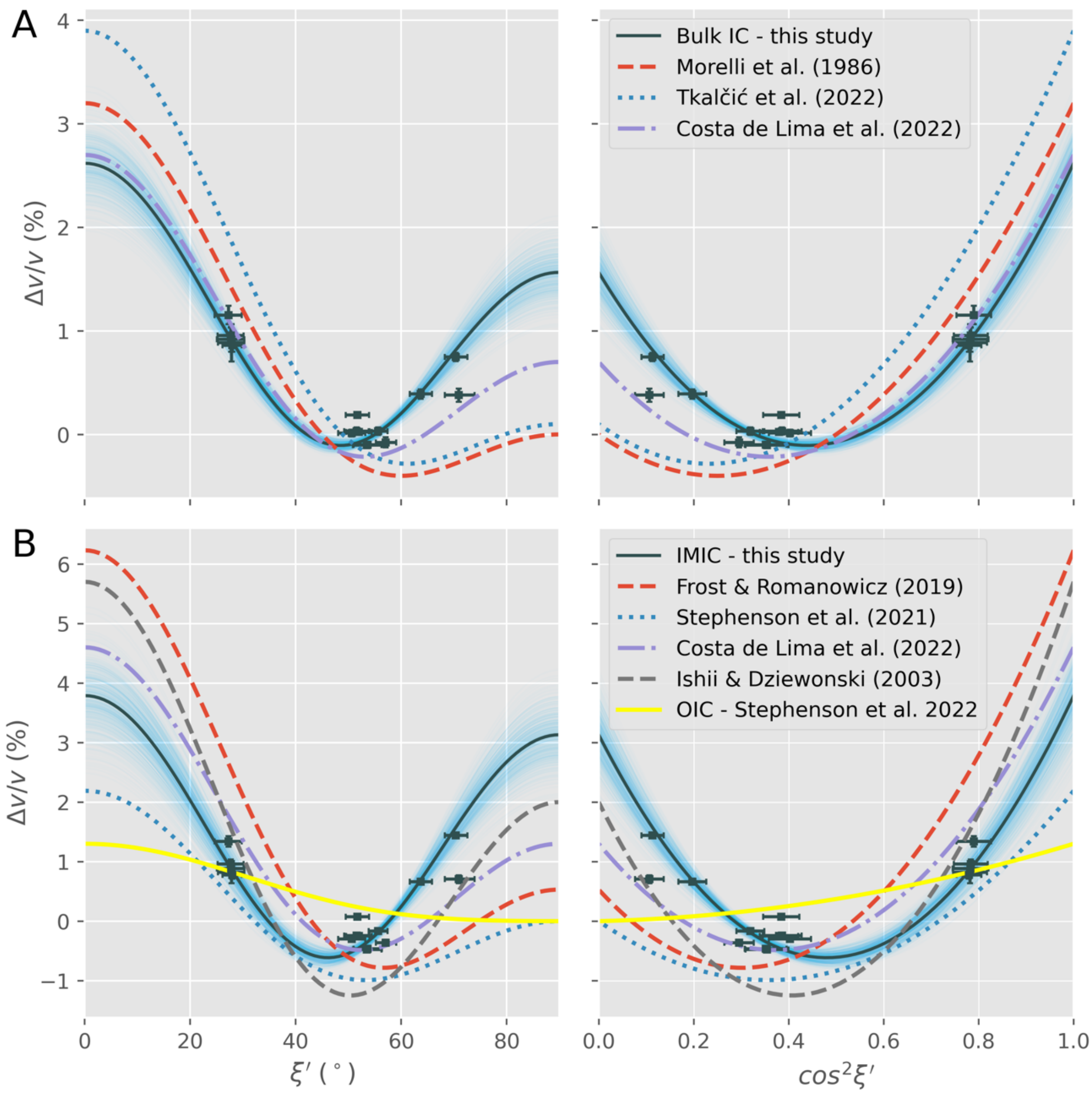

Figure S6. Cylindrically anisotropic model of Earth's inner core inferred from exotic PKIKP multiples. In the left-hand-side panel, measurements and anisotropic models are plotted as a function of ξ, the angle of the ray path to the rotation axis. The mathematically convenient dependent variable, $\cos^2 \xi$, is used on horizontal axes in the right-hand-side panels. In all panels, differential residual measurements, where mantle heterogeneities are corrected for using the LLNL_G3Dv3 model (Simmons et al., 2012), with associated uncertainties, are plotted by dark squares with error bars. Dark solid lines are the optimal anisotropic models parameterized in Equation 4, and light blue opaque lines represent the uncertainty surrounding the optimal model. Various broken lines show models from previous studies (see the legends). The top

row (A) compares our inferred bulk IC model, while the bottom row (B) compares our inferred IMIC model with its predecessors.

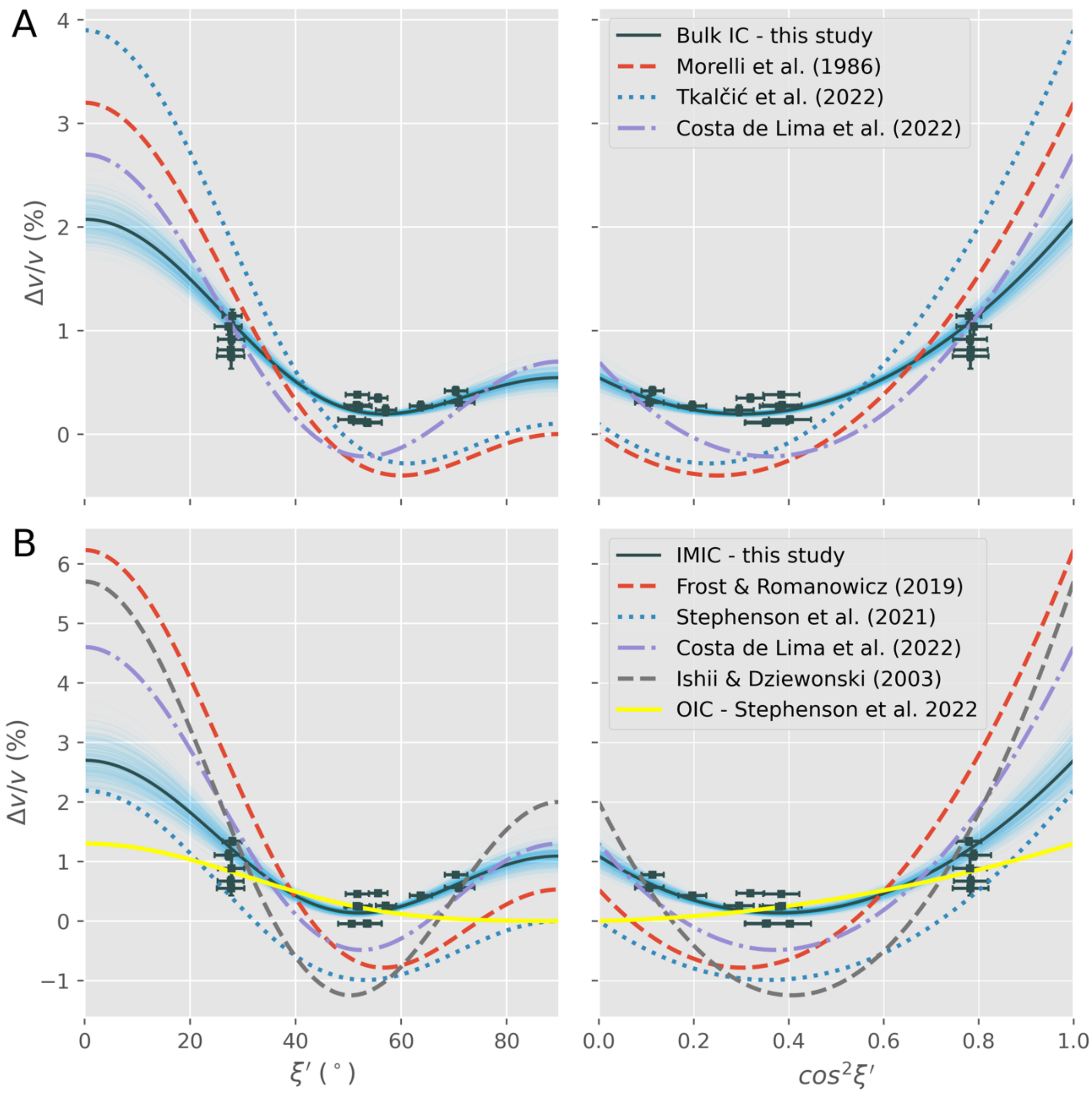

Figure S7. Similar to Figure S6, mantle heterogeneities are corrected for using the MIT-P08 model (C. Li et al., 2008).

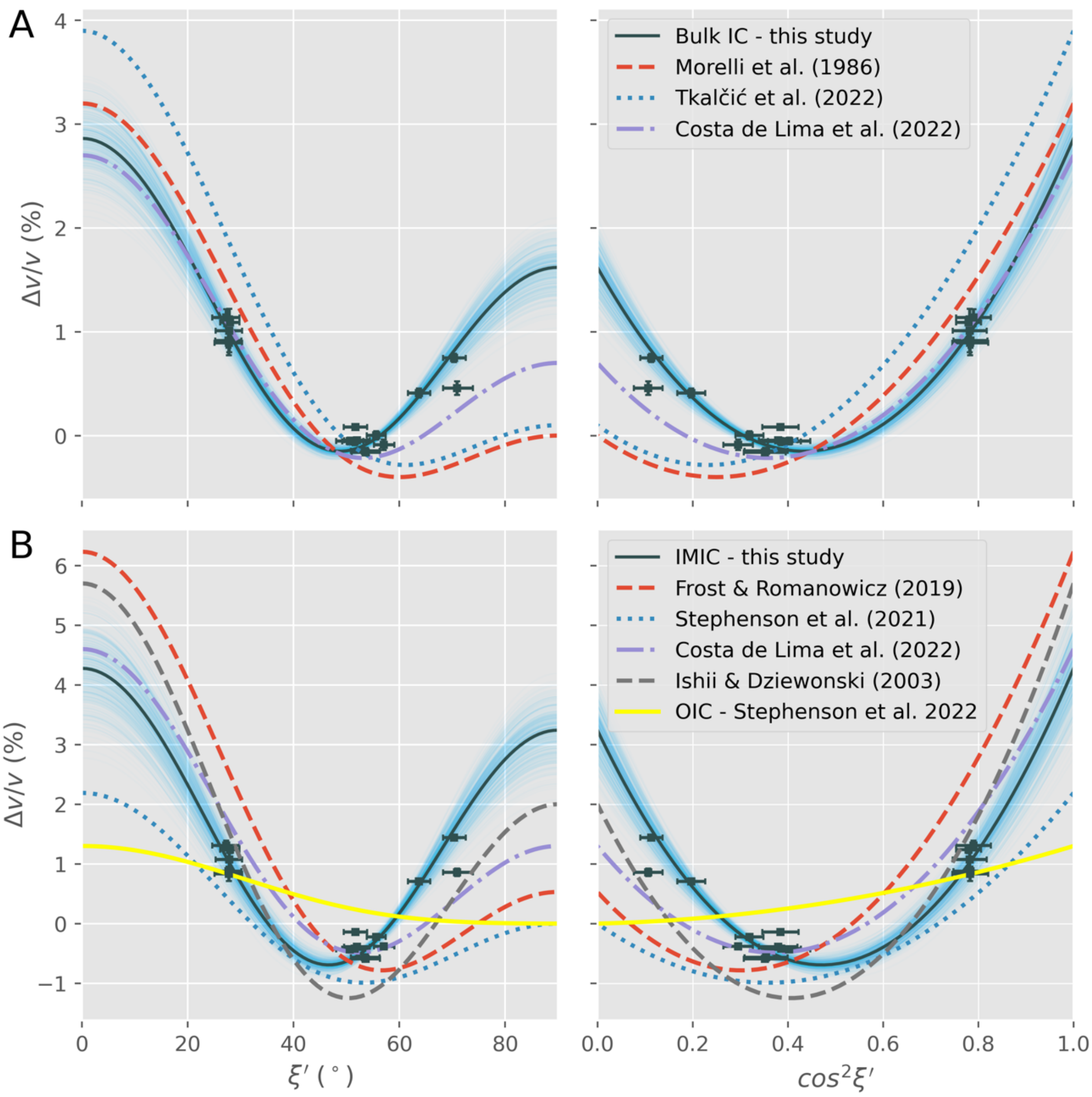

Figure S8. Similar to Figure S6, but mantle heterogeneities are not considered in this case.

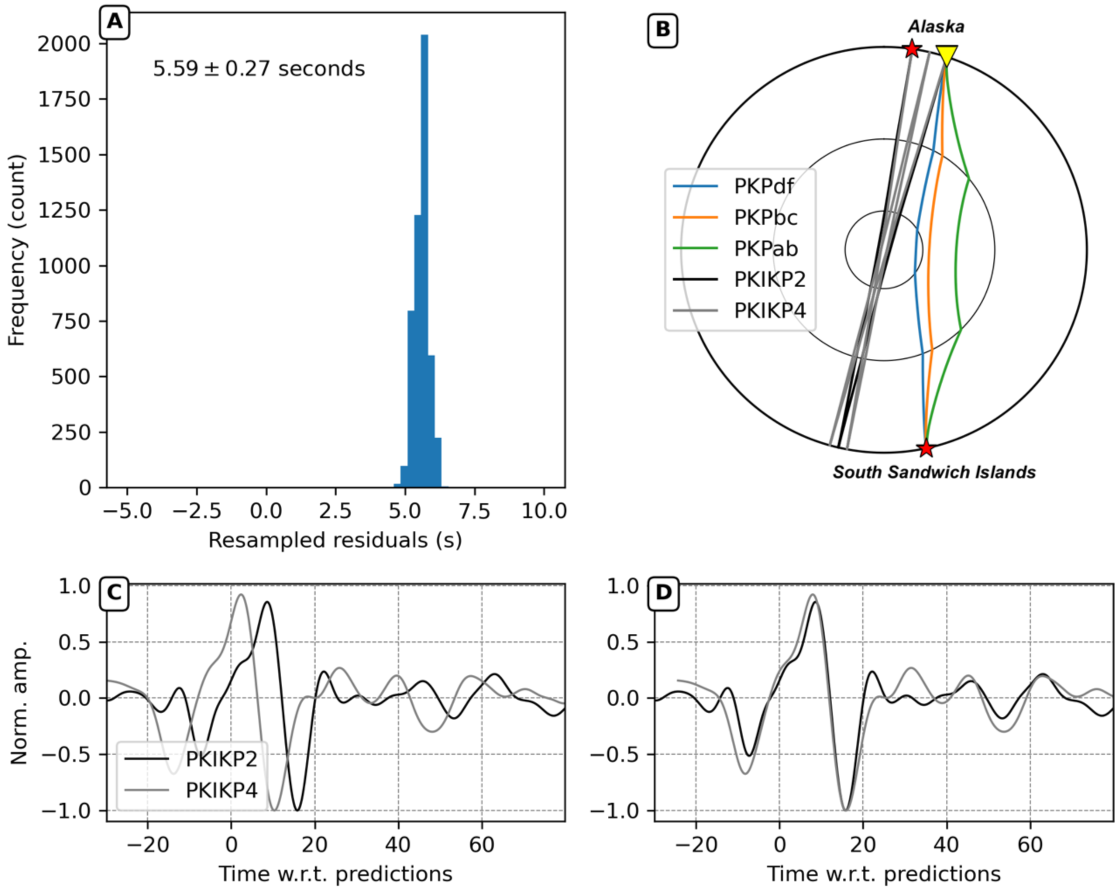

Figure S9. Measuring residual travel times of PKIKP4-PKIKP2 observation for the Mw 7.1 Anchorage earthquake 30/11/2018 by stacked waveform cross-correlation. *A)* The histogram shows the bootstrapping result comprising of residual travel times for 5000 resampled station sets with replacement. The estimated mean and standard deviation are written on the top left. *B)* The Earth cross-section compares how the IC is sampled by the exotic PKIKP multiples with 'traditional' PKP waves along the SSI-Alaska path. *C)* The stacked PKIKP2 and PKIKP4 waveforms are plotted using their actual times with respect to the theoretical predictions. *D)* Similar to C) but the stacked PKIKP4 waveform is aligned to PKIKP2 by the estimated mean residual.

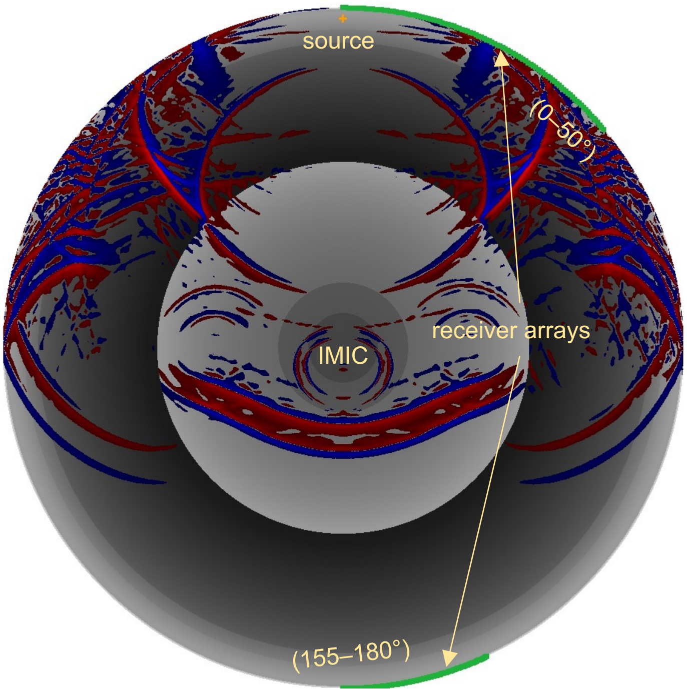

Figure S10. A snapshot at 650 seconds of the specfem2d (e.g., Komatitsch et al., 2002) simulation in an Earth's cross-section. A 200-km-deep seismic source is marked by an orange cross; podal and antipodal seismic arrays are denoted by green triangles. Negative and positive z-velocity components are plotted in blue and red colors. A 650-km-radius IMIC is characterized by an increase from the ak135 background model.

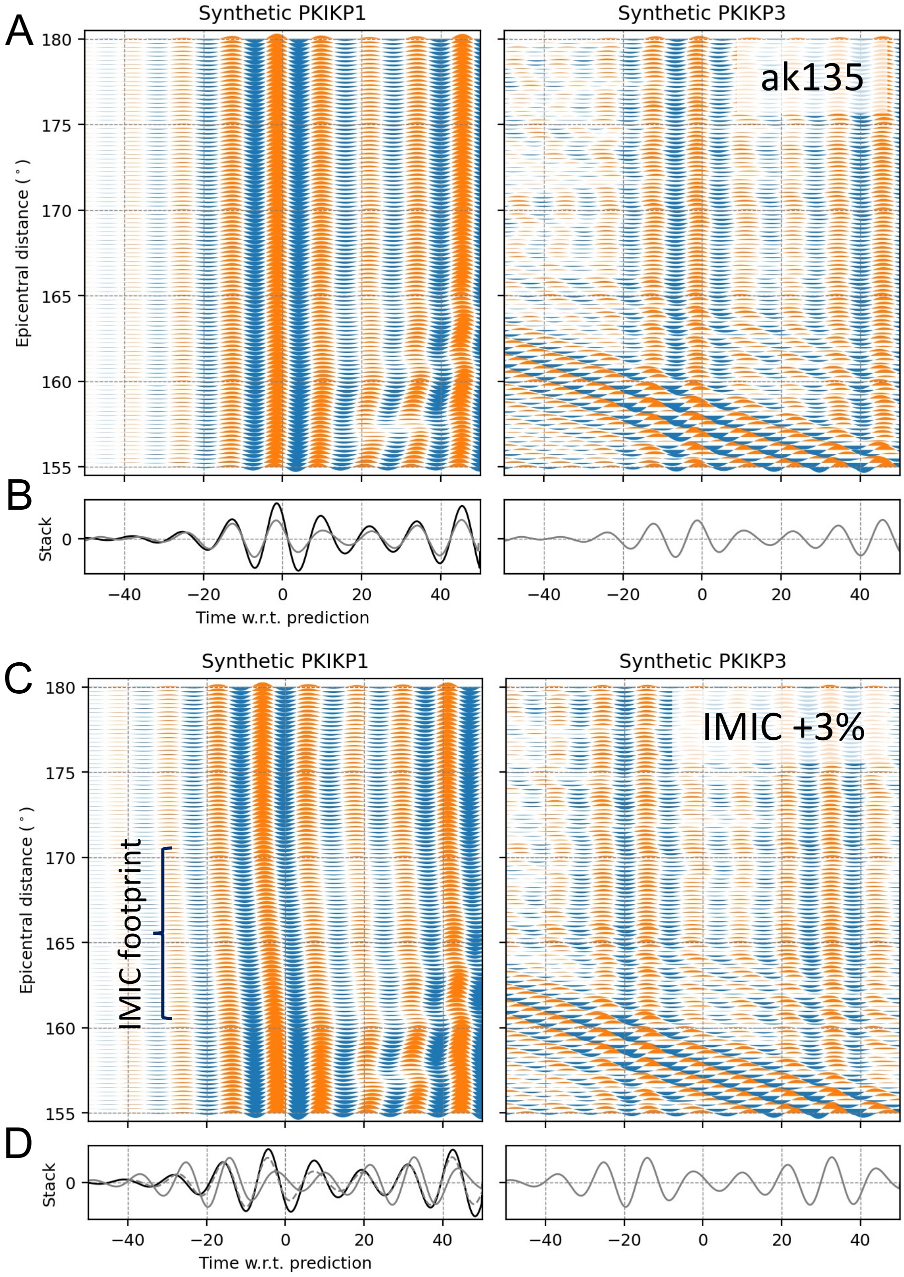

Figure S11. *A)* Individual synthetic waveforms of PKIKP and PKIKP3 (bandpass-filtered in the band 7–13 s) are aligned with respect to their ray-theoretical predictions. The main arrivals are located near 0 s, while the depth reflections are seen ~43 s corresponding to a 200-km-deep source. We use the ak135 model (Kennett et al., 1995) for both the computational domain and travel time prediction. *B)* Black (PKIKP) and gray (PKIKP3) waveforms are stacked from the individual waveforms in A). Stacked PKIKP3 waveform (gray) is plotted in both left and right panel. *C)* and *D)* are similar to A) and B) but for the model with an IMIC P-wave speed increased by 3%.

The stacked PKIKP3 waveform (gray dashed line) in the left panel of D) is identical to that in E) but shifted to align with the stacked PKIKP1 waveform (black line), because PKIKP3 waves arrive earlier than predicted due to the faster IMIC in this model.

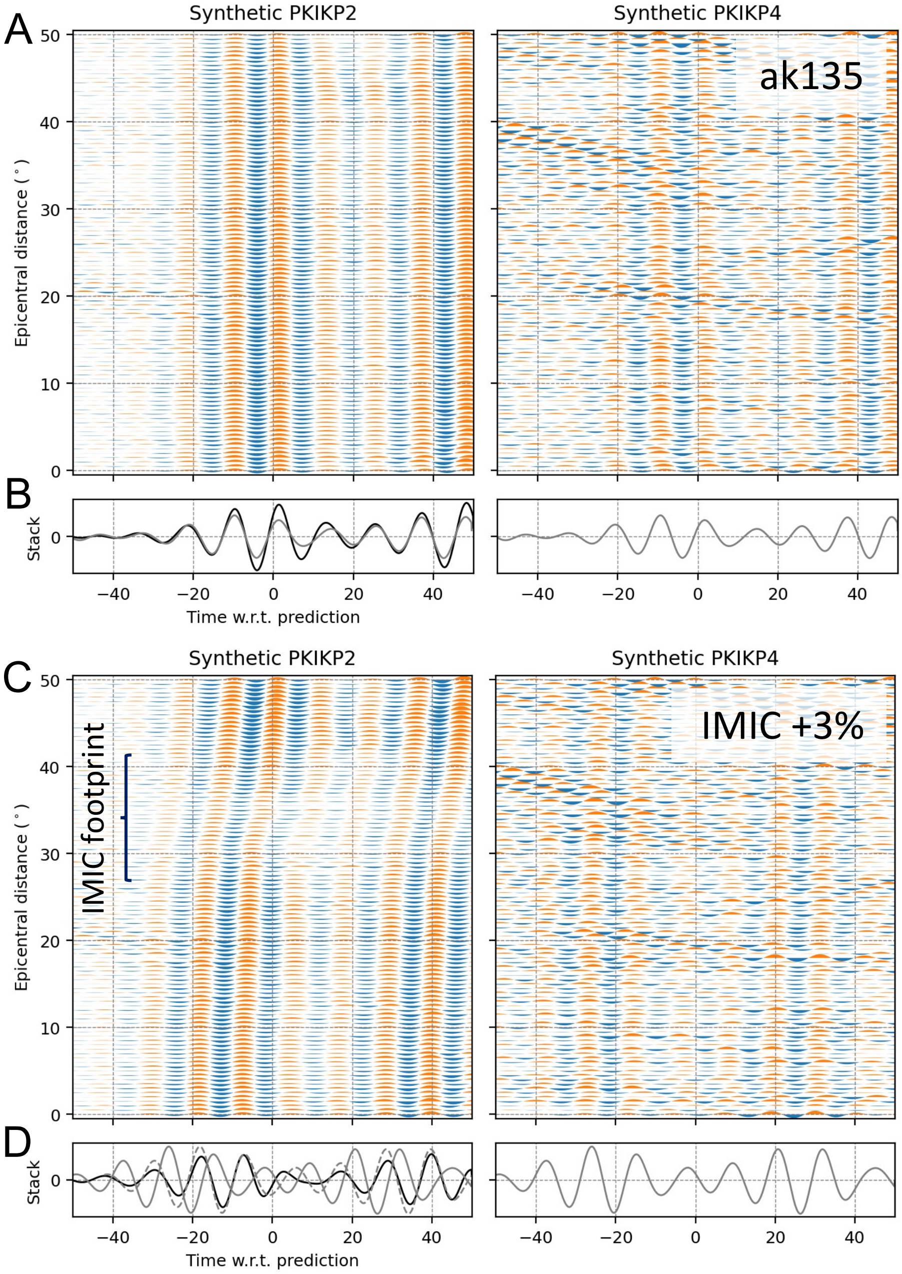

Figure S12. Similar to Figure S11, but for the PKIKP2 and PKIKP4 phases.

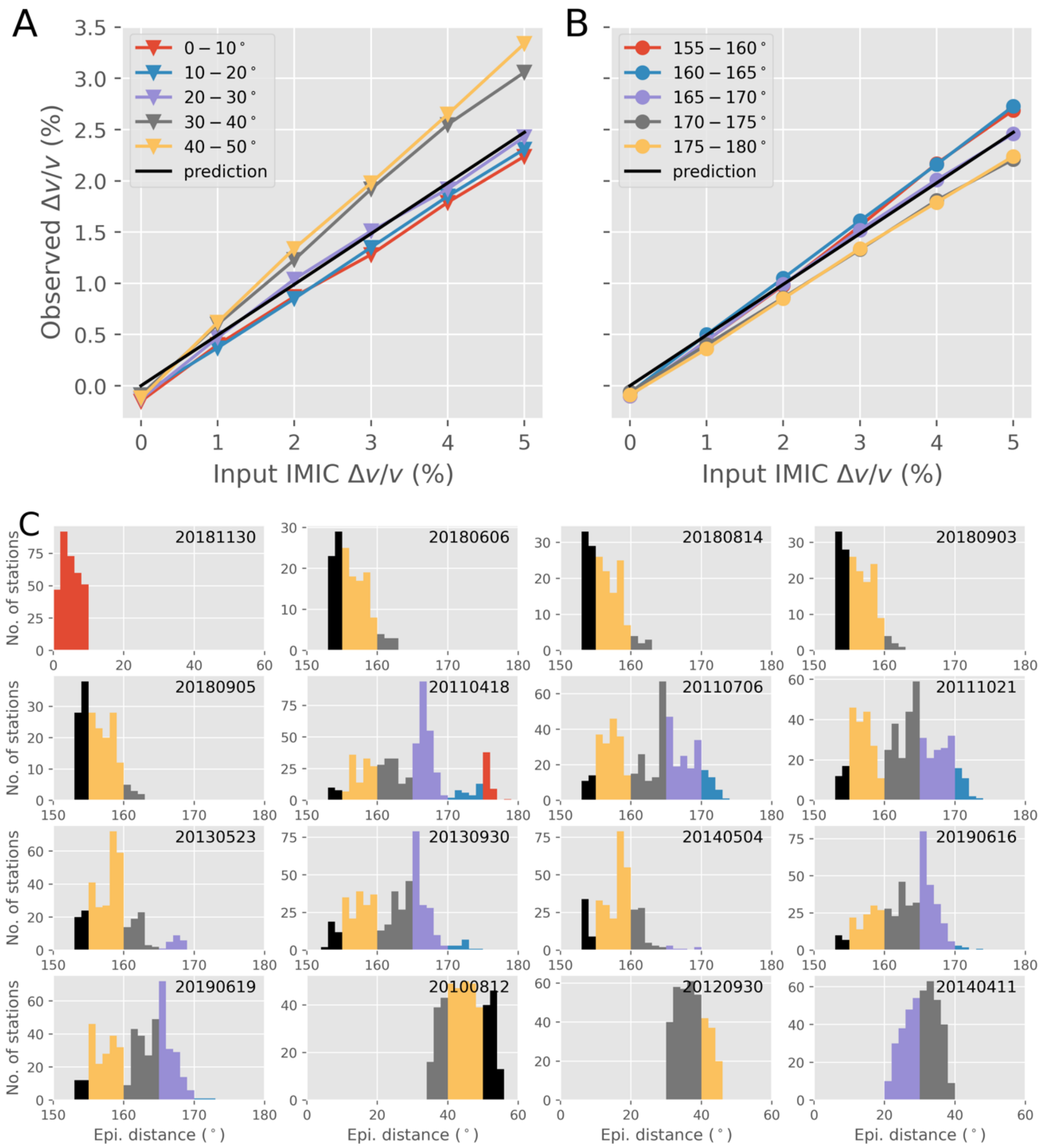

Figure S13. Method sensitivity test to recover the increased P-wave speed confined in a 650-km-radius IMIC using PKIKP4-PKIKP2 pair (panel A) and PKIKP3-PKIKP1 pair (panel B). The horizontal axes represent a synthetic input increase in the IMIC's P-wave speed, $\Delta v/v$. Vertical axes represent the IC-average P-wave speed increases obtained by cross-correlating synthetic waveforms in five distance brackets (see the legends) in the same way the observations are made. The black solid lines show the predicted (theoretical) values corresponding to the inputs. C) Sub-panels show the distribution of epicentral distances corresponding to 16 residual measurements (see

Figure S4). The histograms are colored by distance brackets in the same convention as the above panels.

Supplementary Figures

Event 20100812; Mantle correction model: DETOX-P3

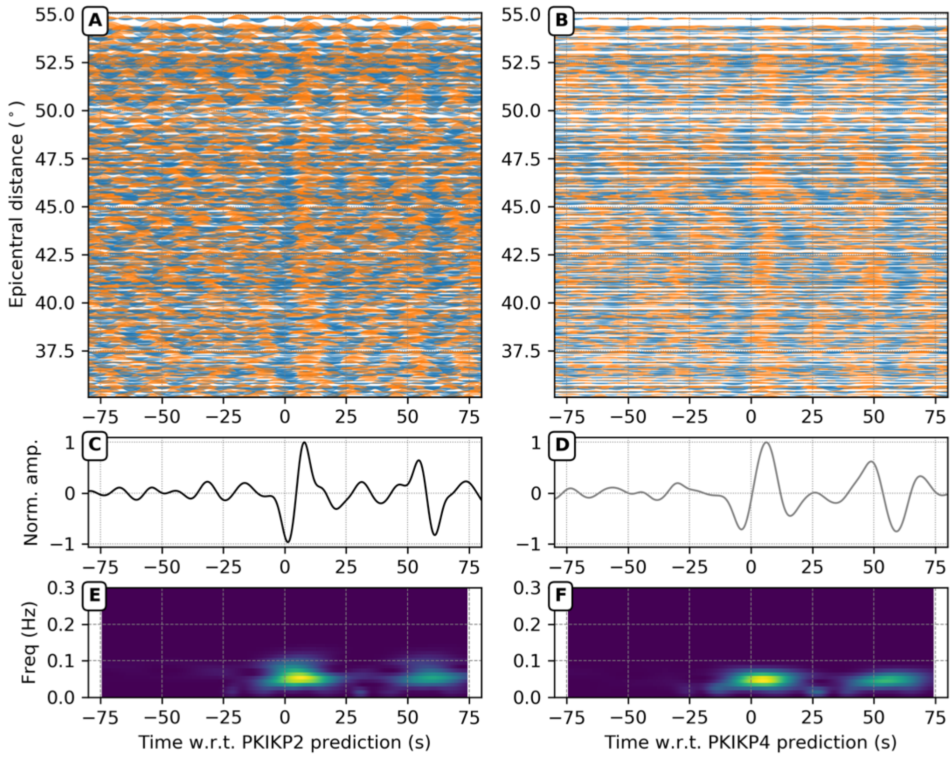

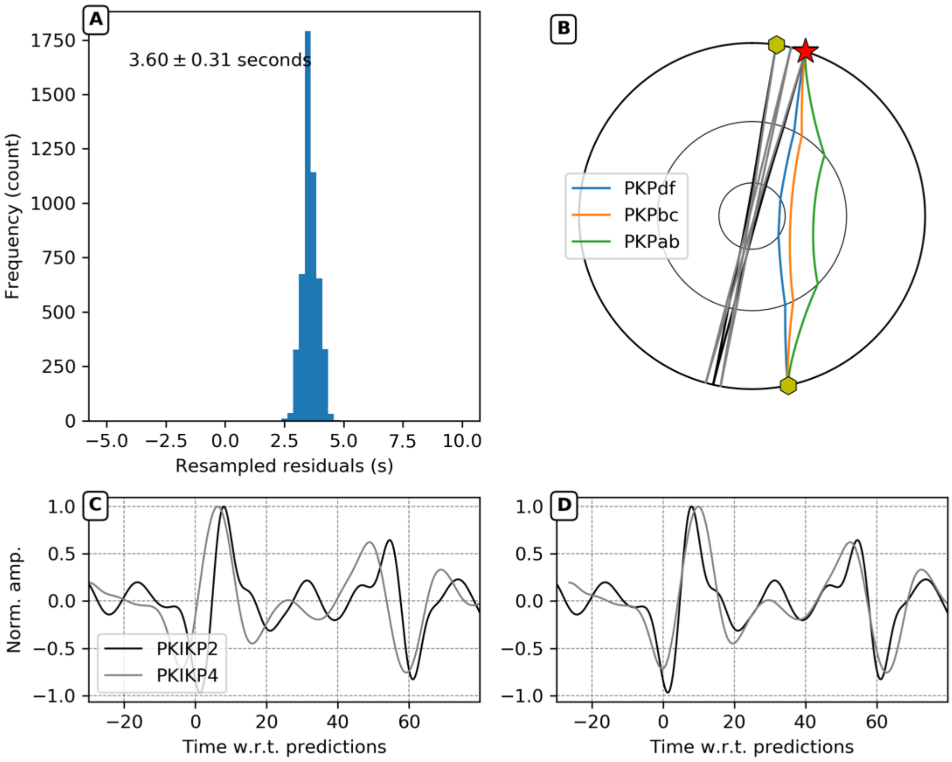

Event 20110418; Mantle correction model: DETOX-P3

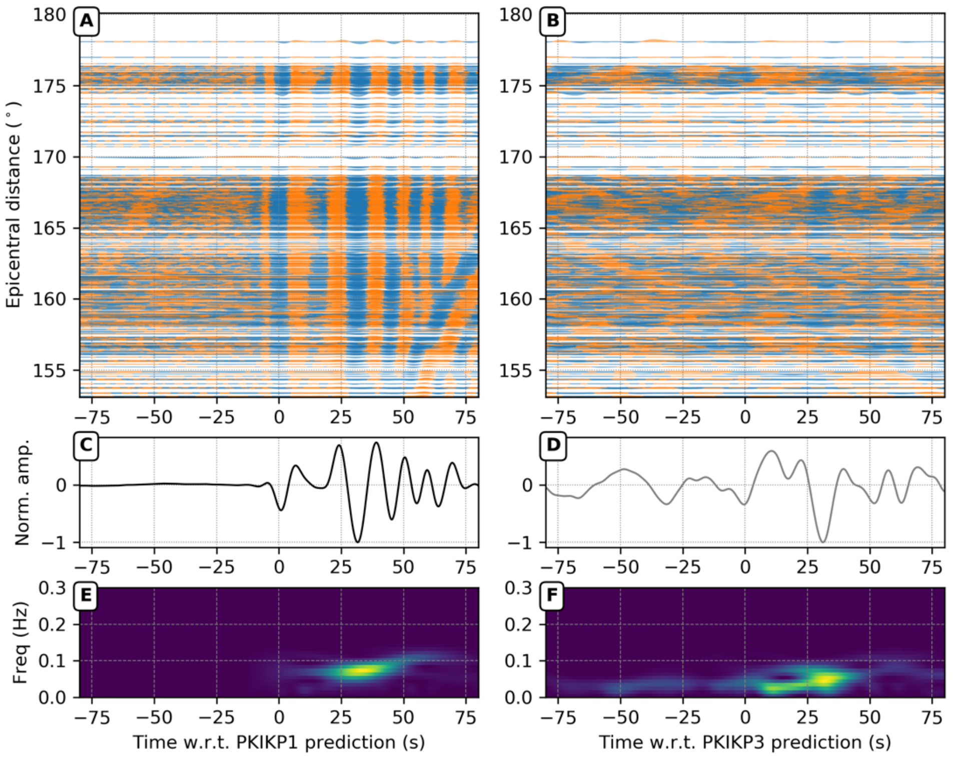

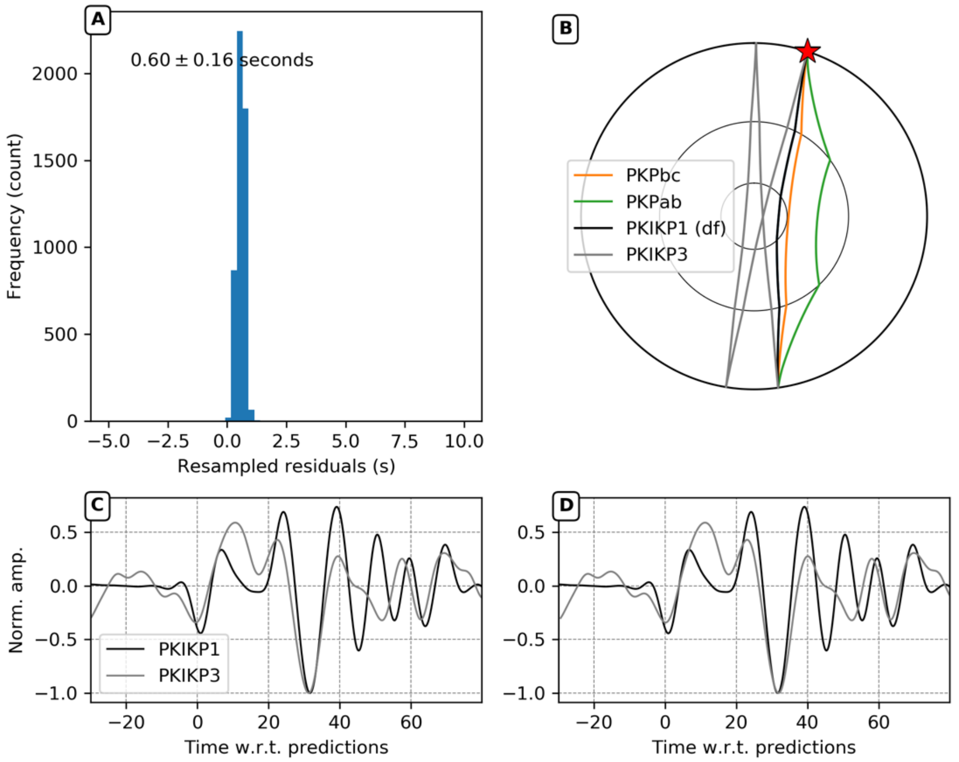

Event 20110706; Mantle correction model: DETOX-P3

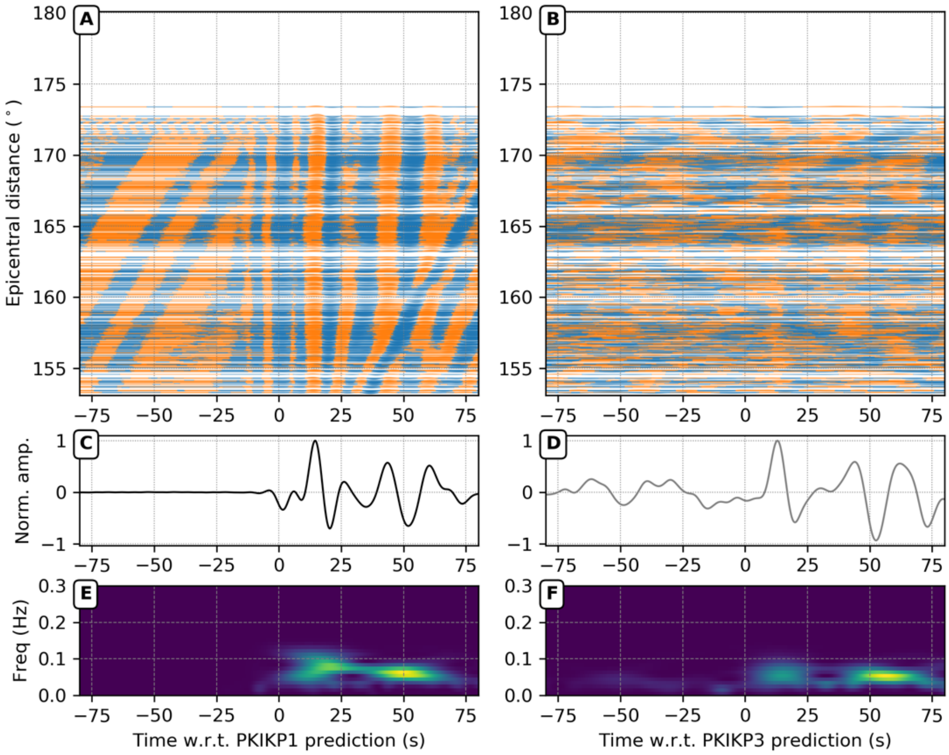

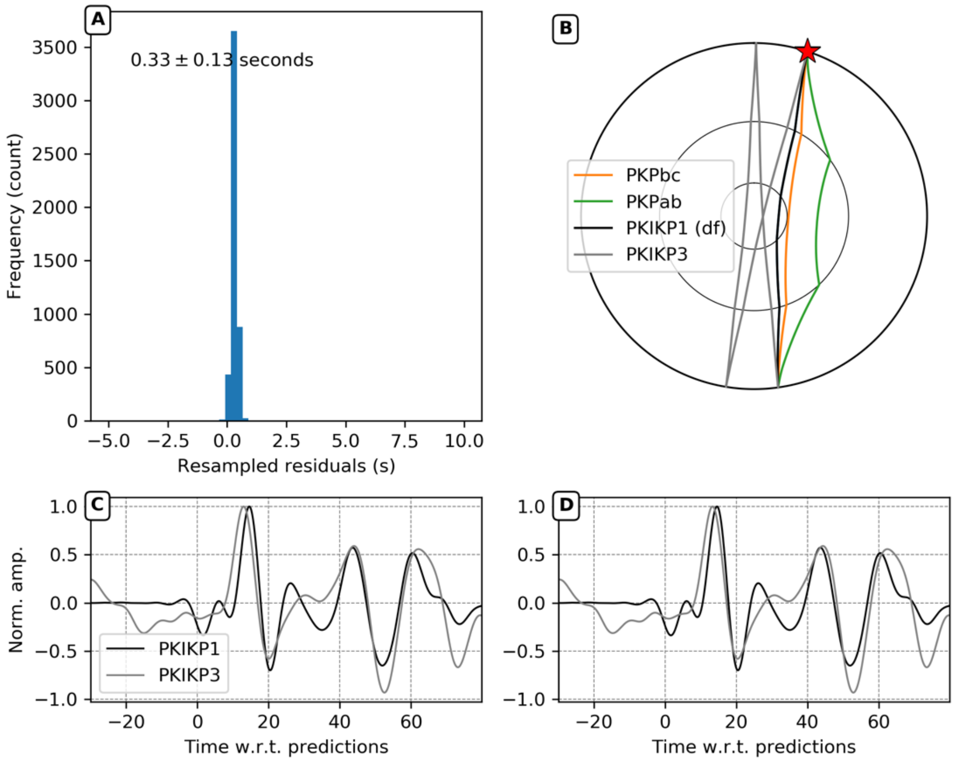

Event 20111021; Mantle correction model: DETOX-P3

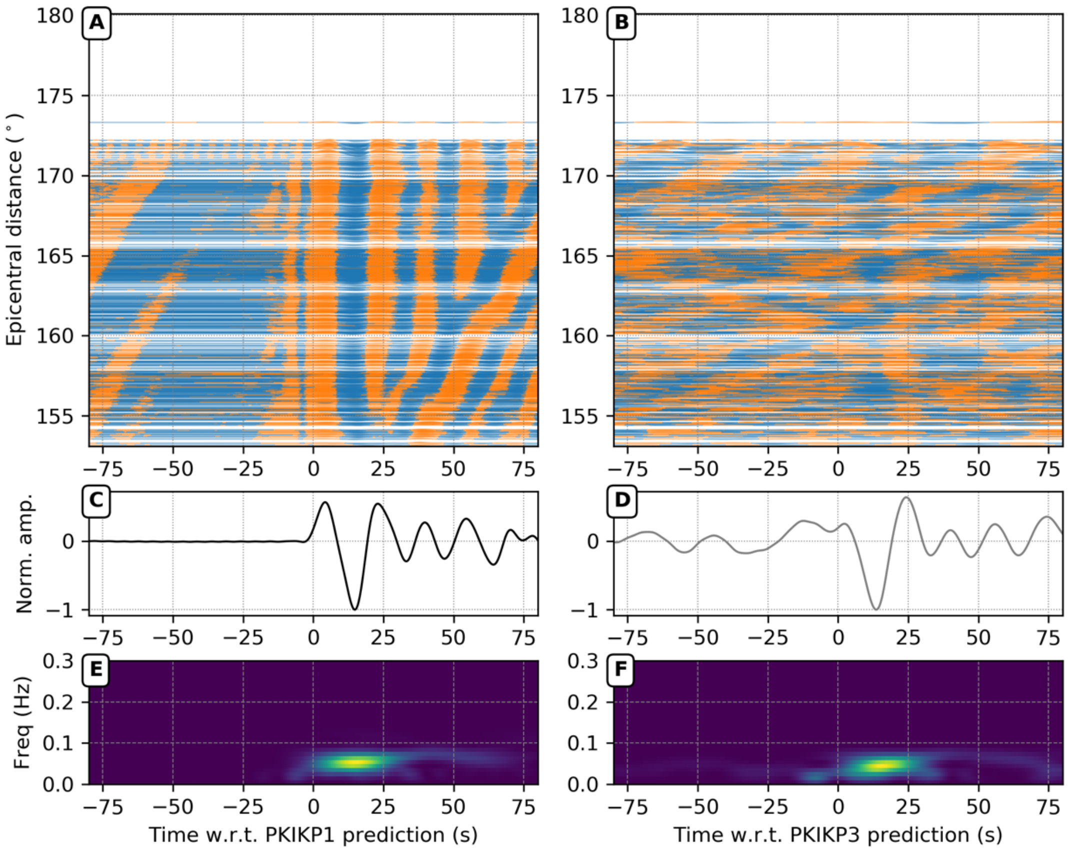

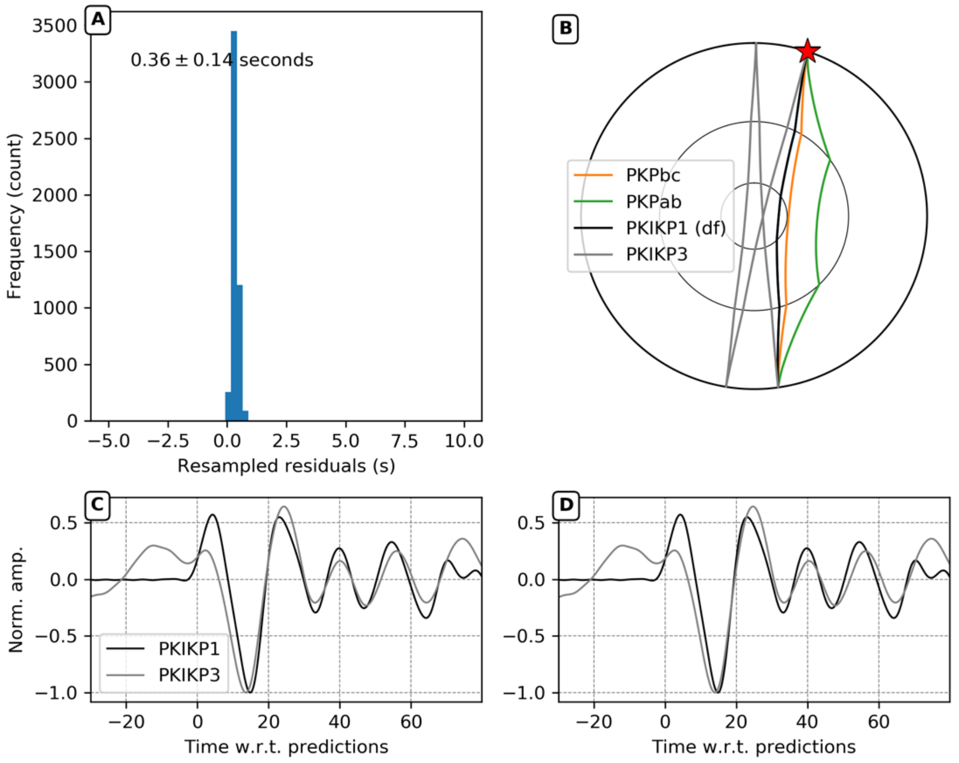

Event 20120930; Mantle correction model: DETOX-P3

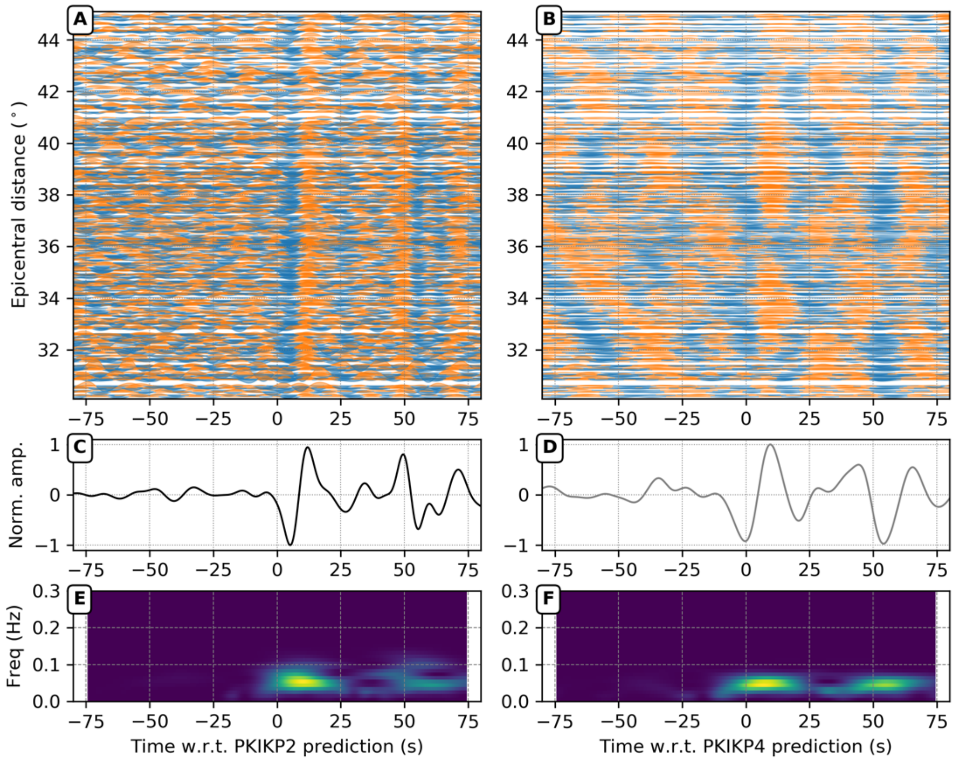

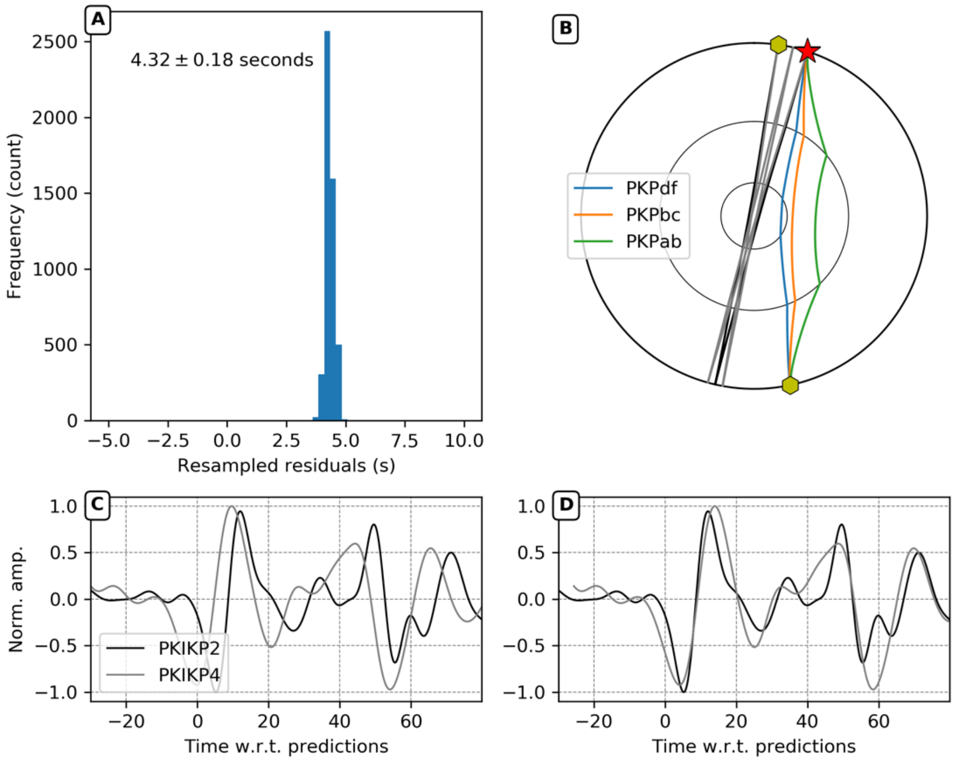

Event 20130523; Mantle correction model: DETOX-P3

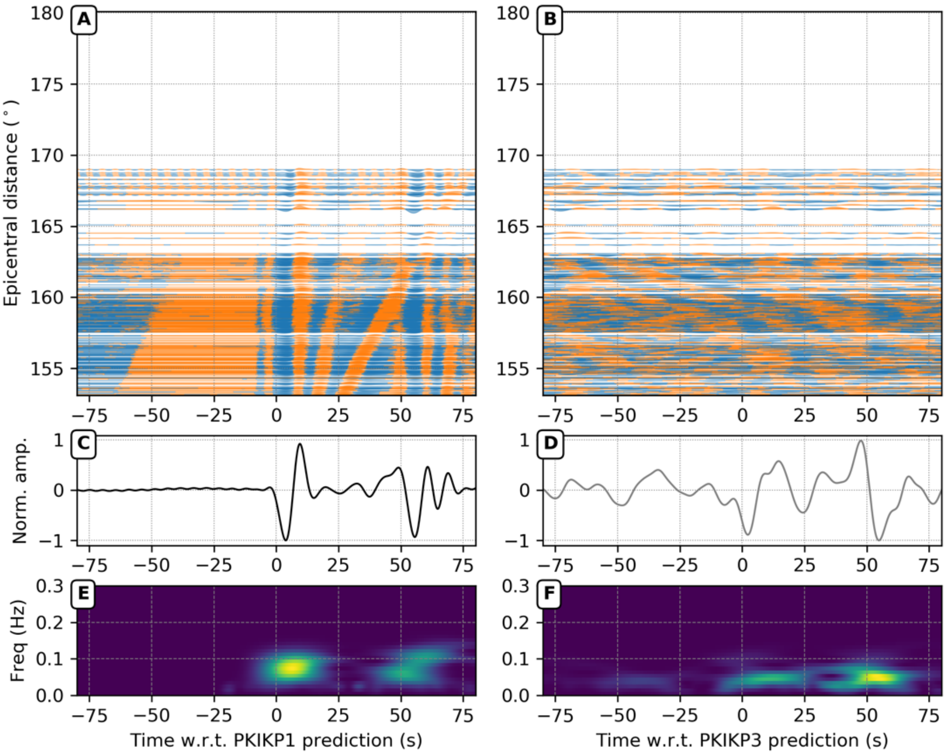

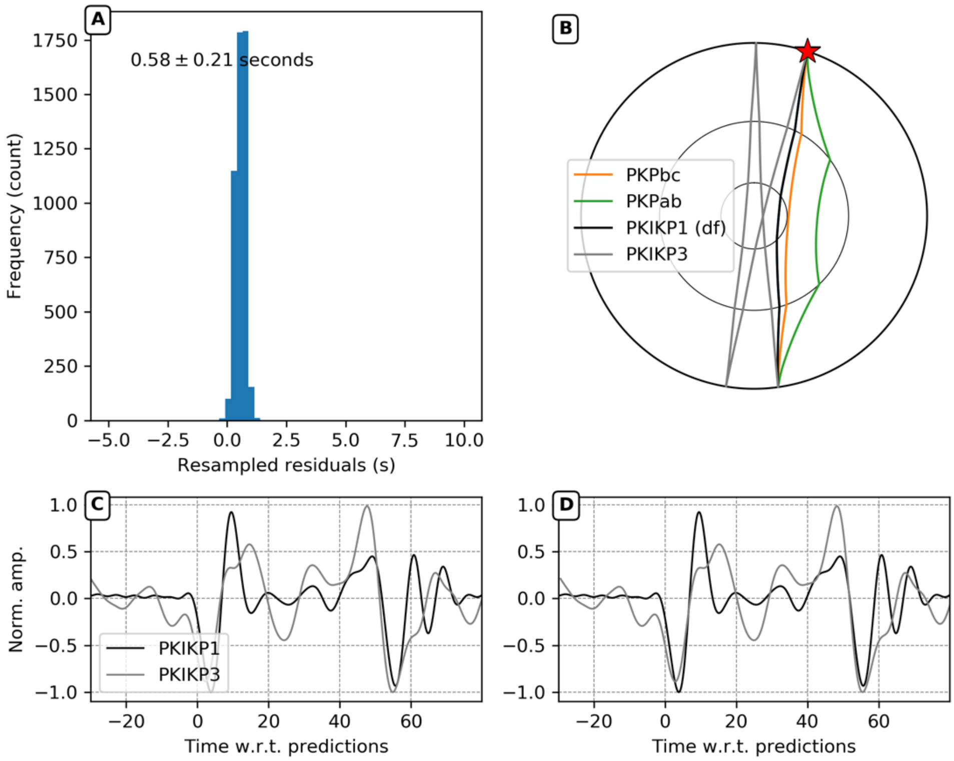

Event 20130930; Mantle correction model: DETOX-P3

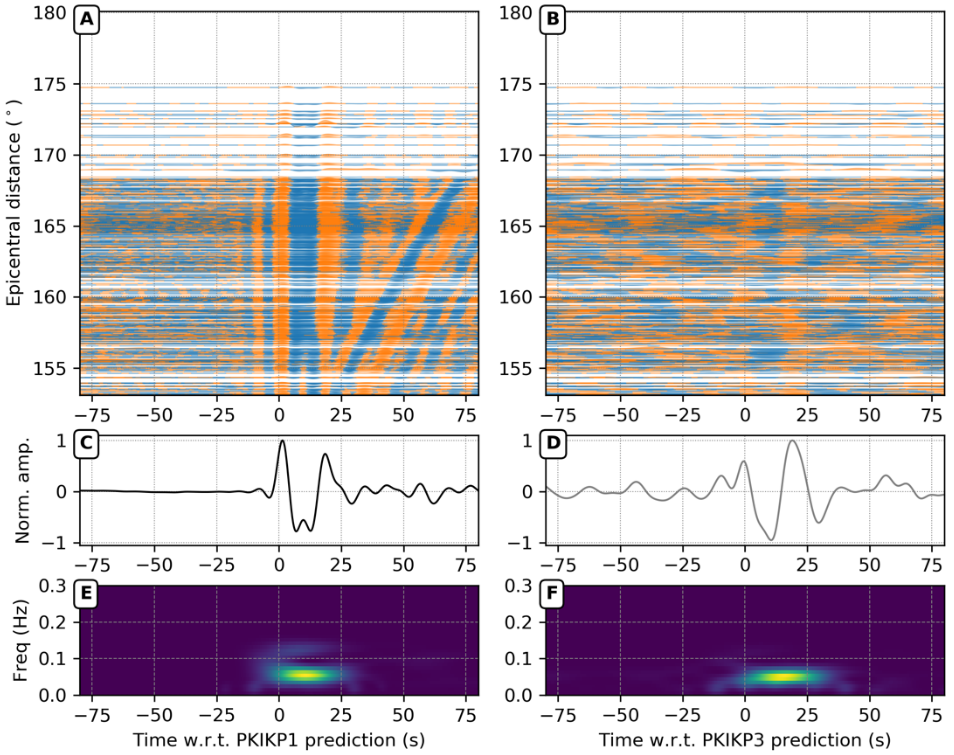

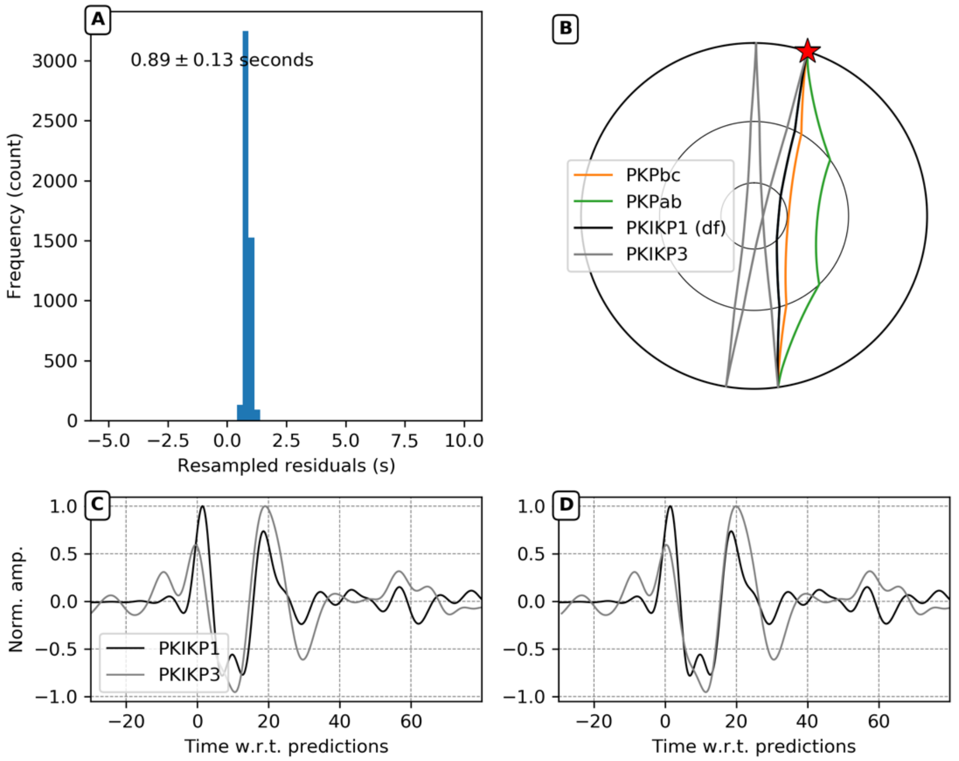

Event 20140411; Mantle correction model: DETOX-P3

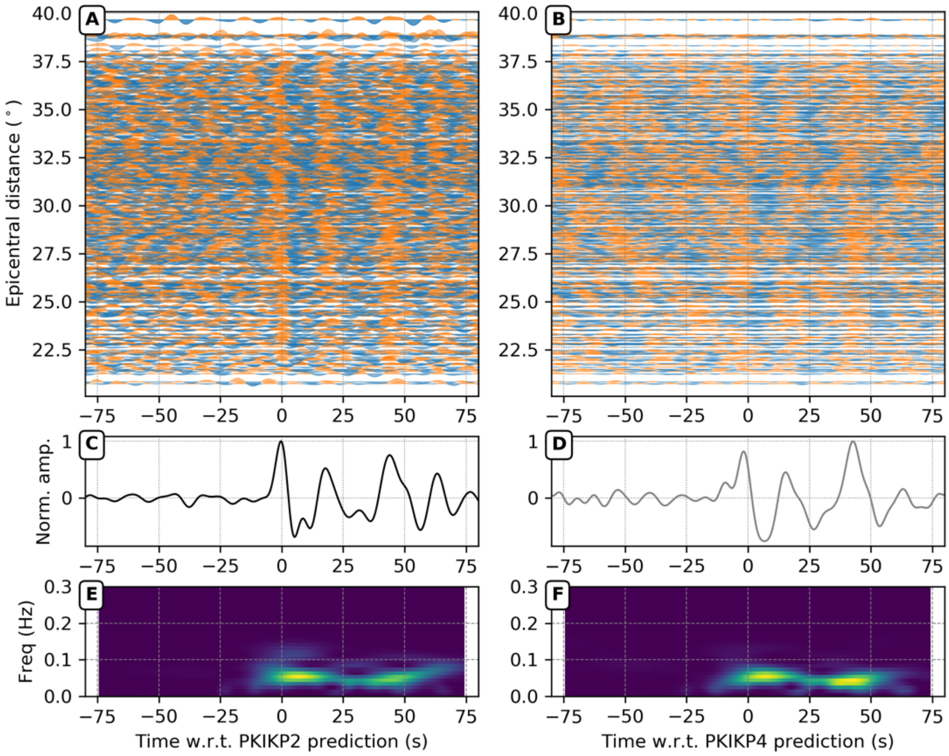

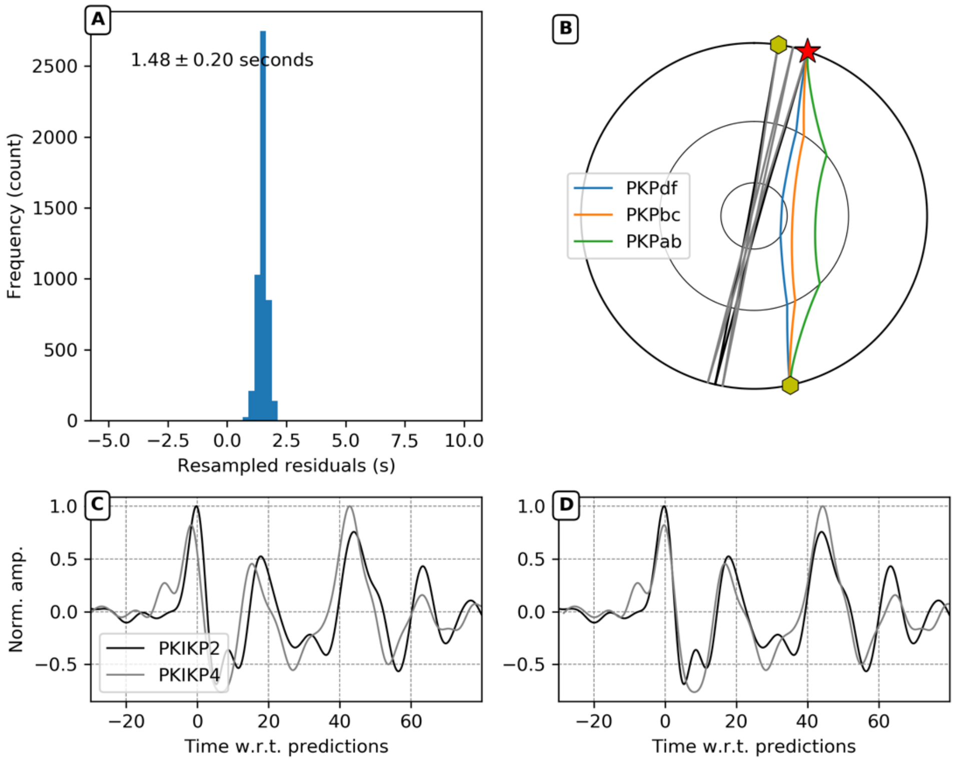

Event 20140504; Mantle correction model: DETOX-P3

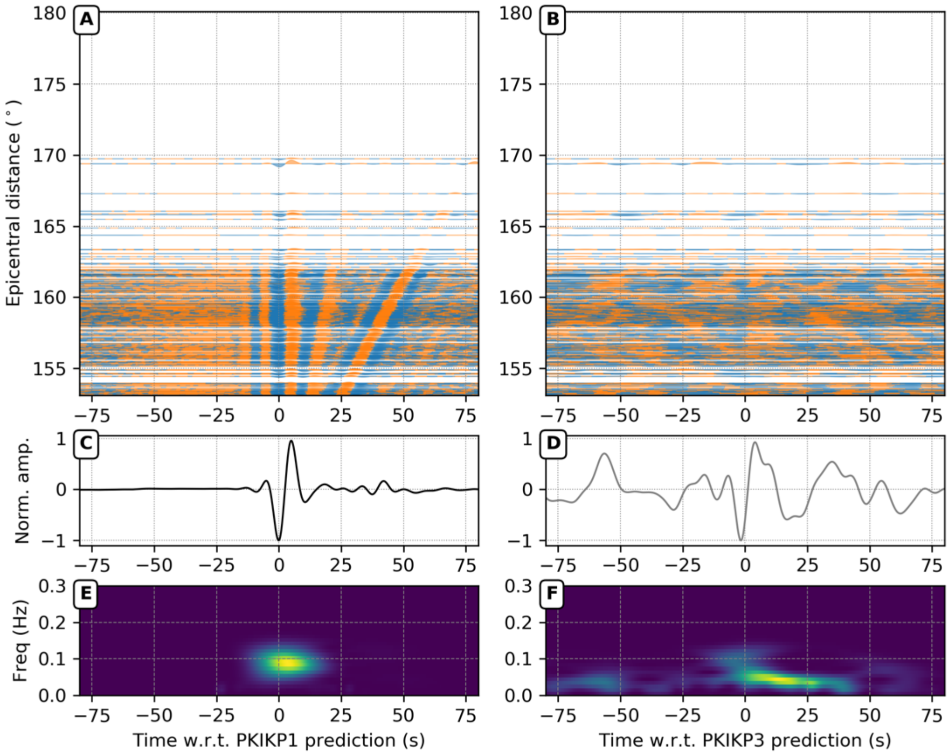

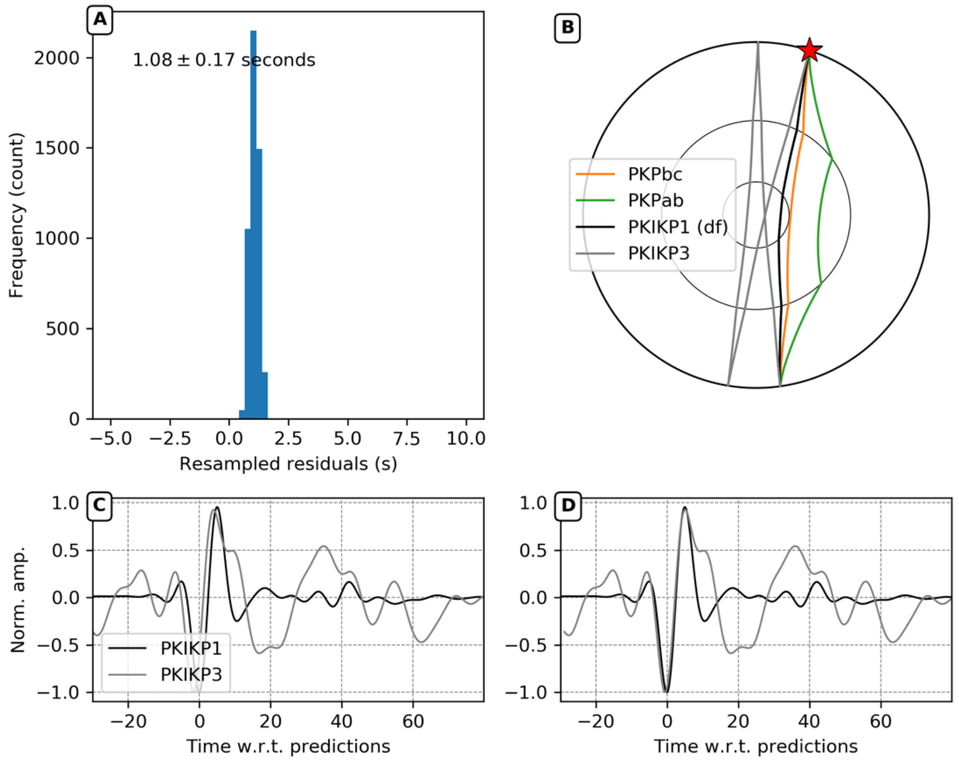

Event 20180606; Mantle correction model: DETOX-P3

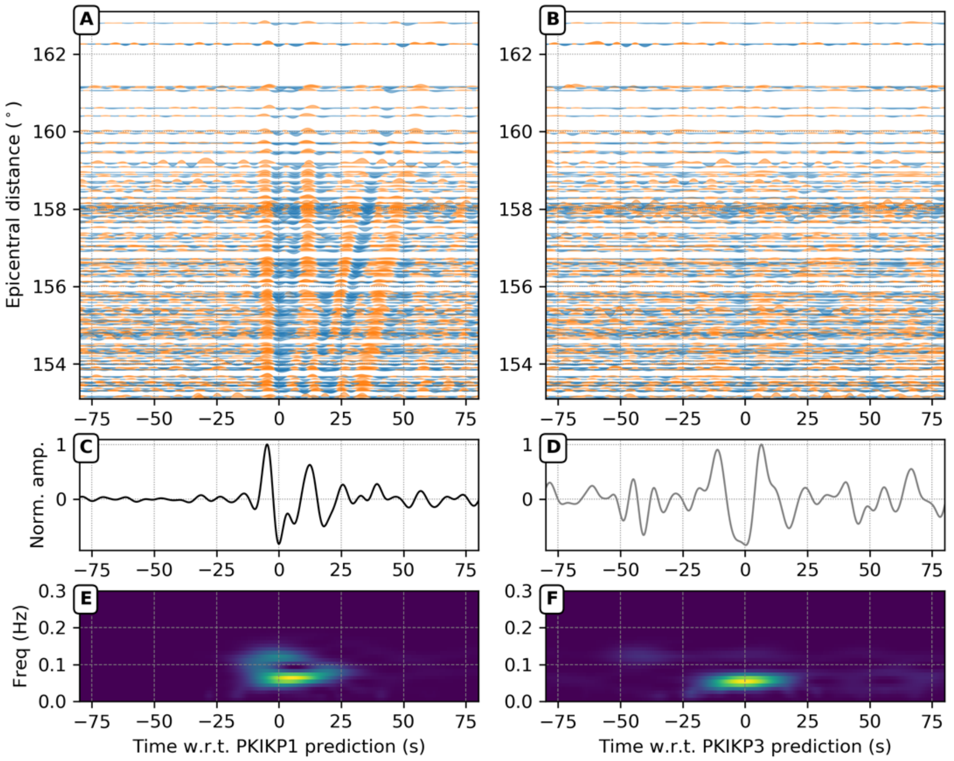

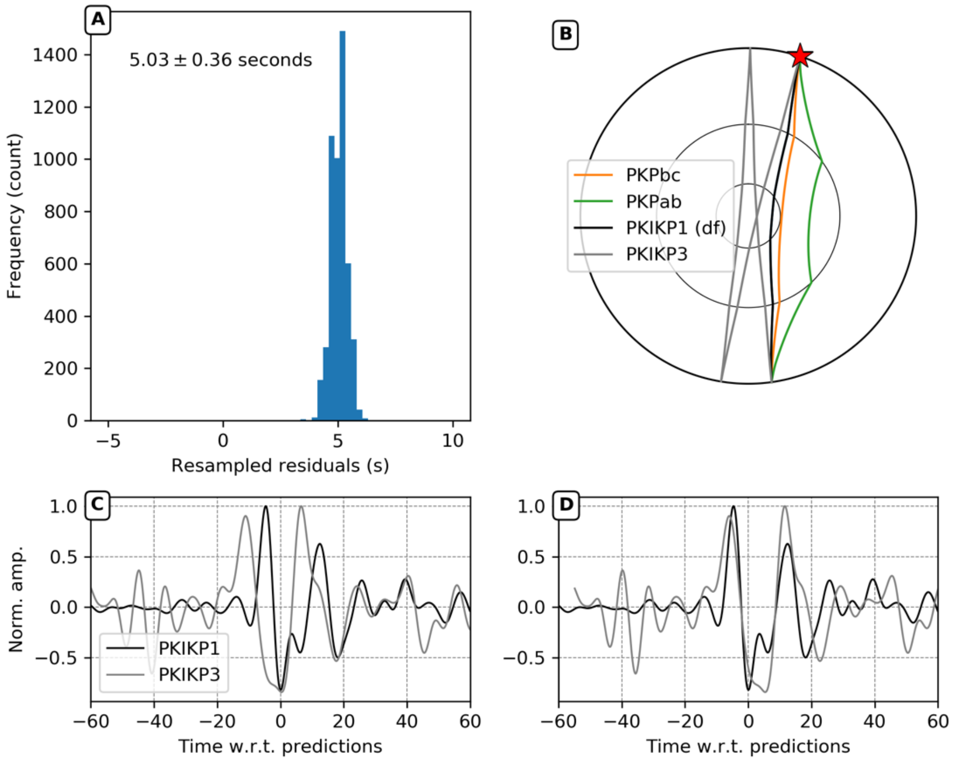

Event 20180814; Mantle correction model: DETOX-P3

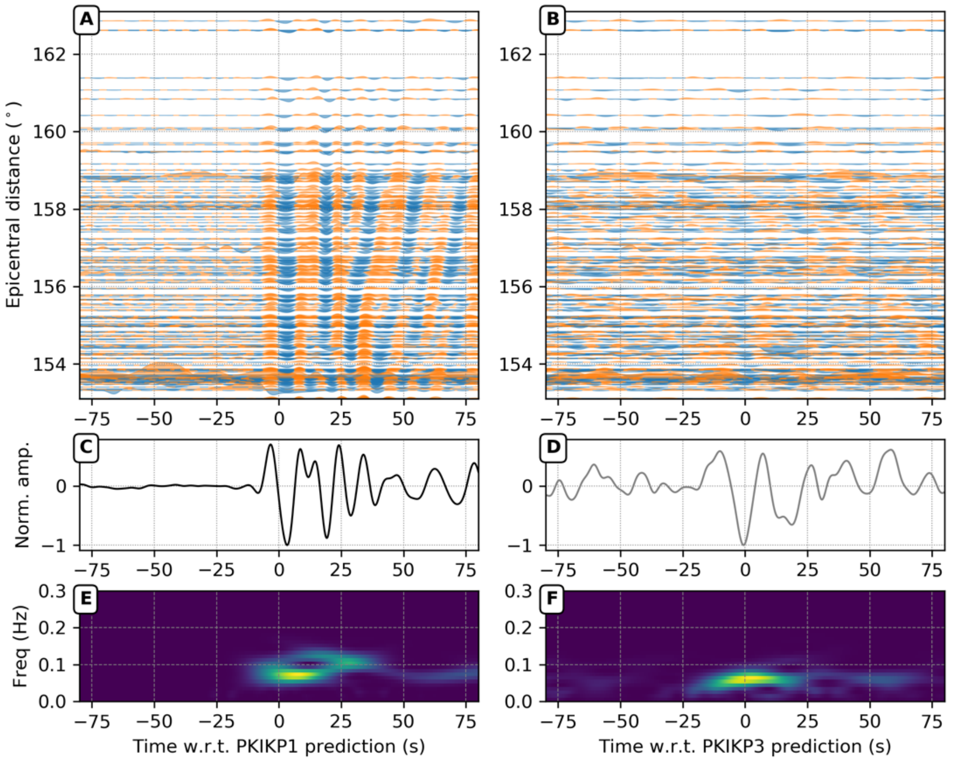

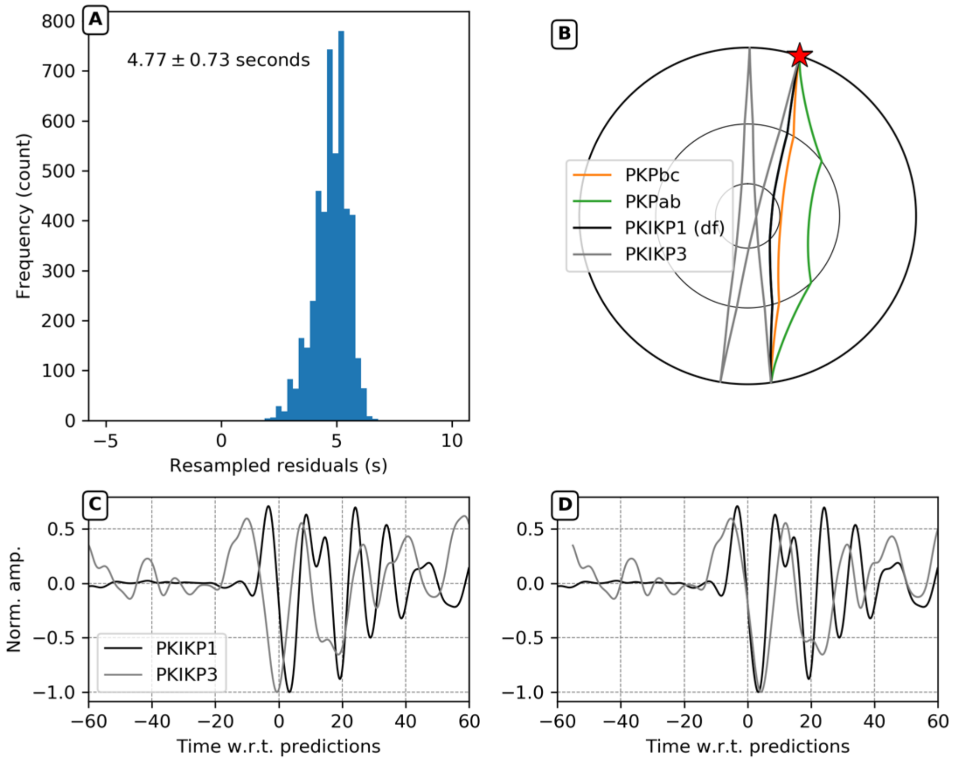

Event 20180903; Mantle correction model: DETOX-P3

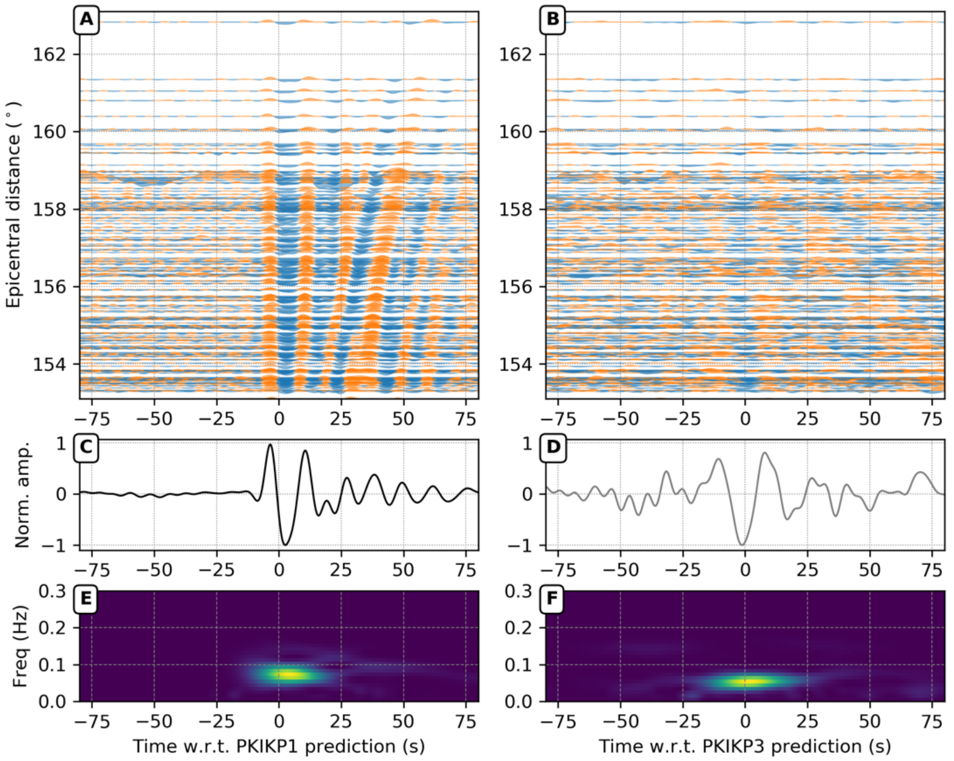

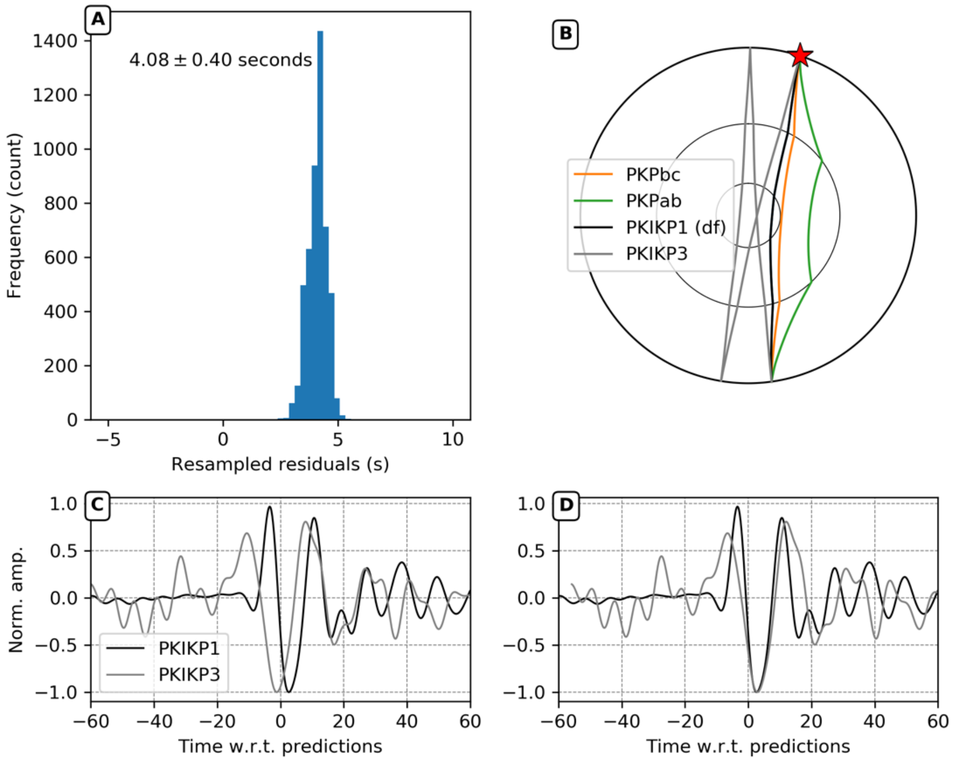

Event 20180905; Mantle correction model: DETOX-P3

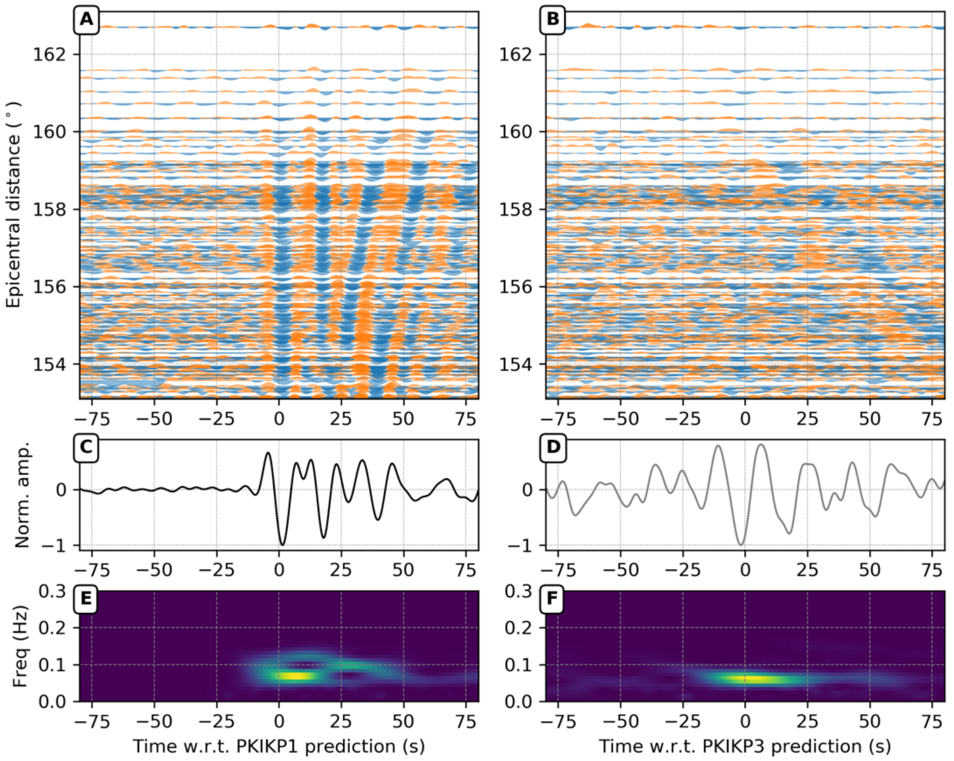

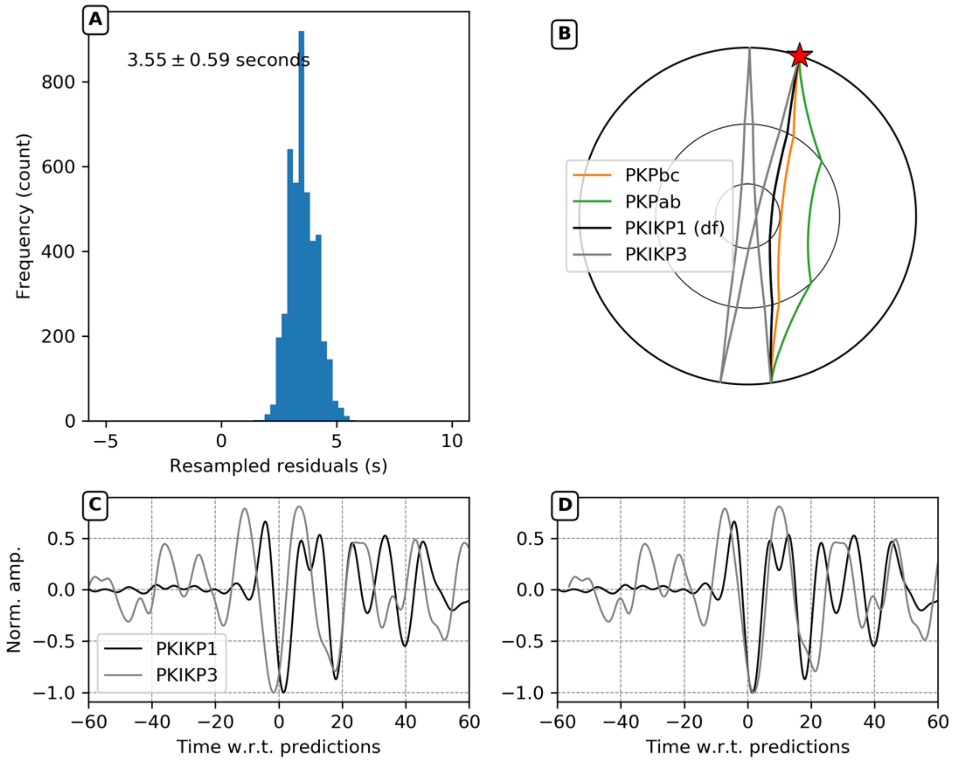

Event 20181130; Mantle correction model: DETOX-P3

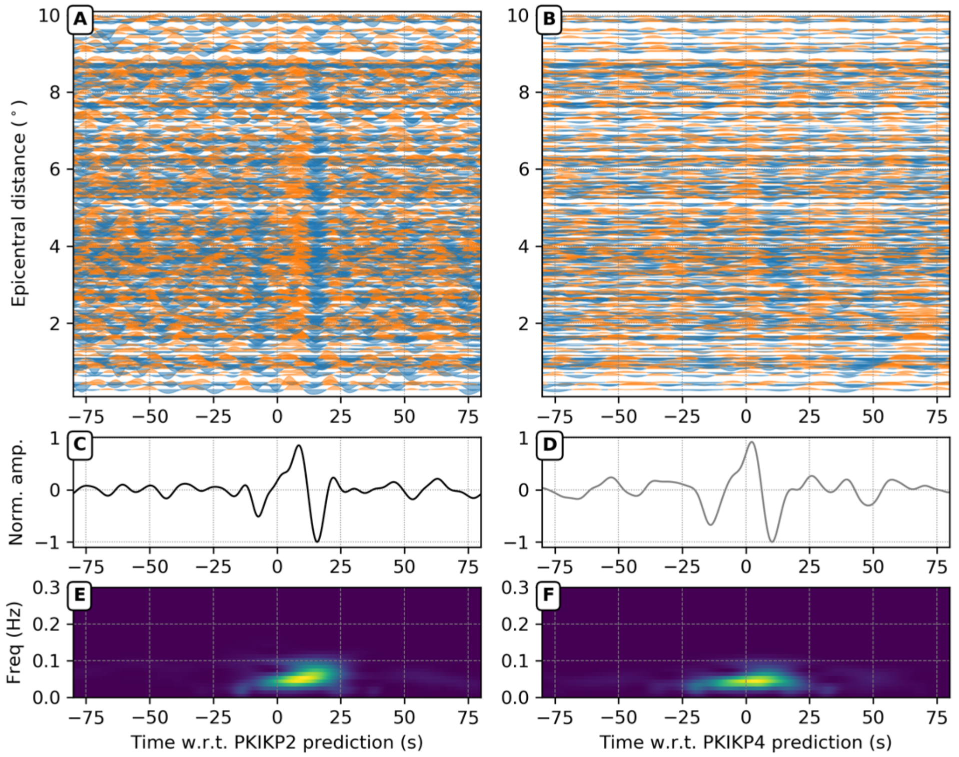

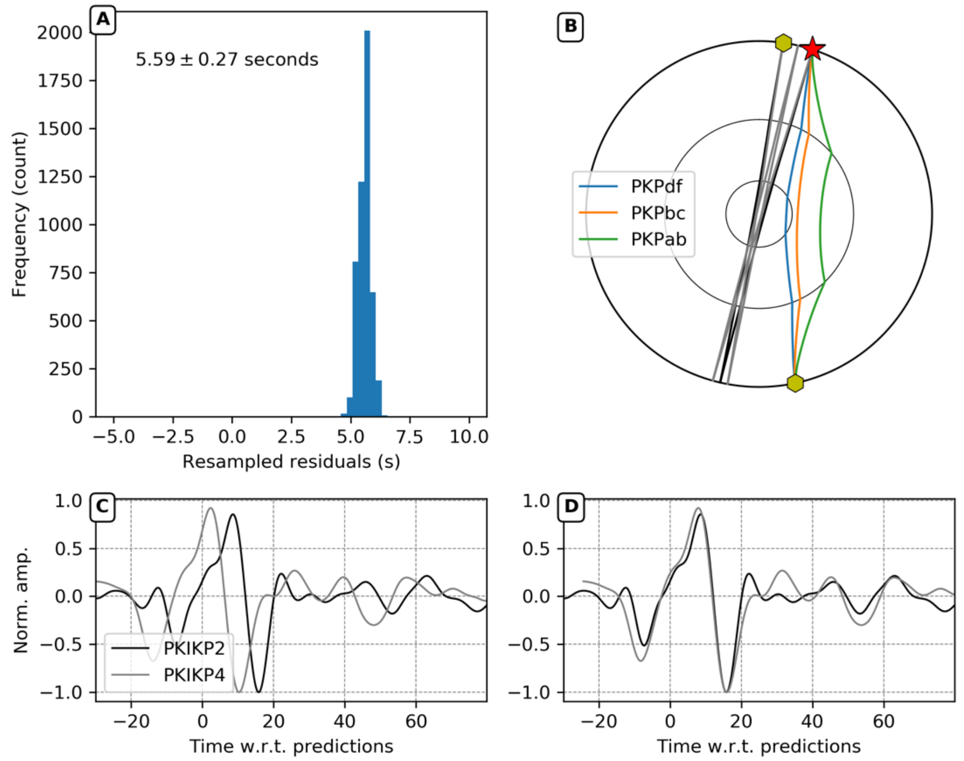

Event 20190616; Mantle correction model: DETOX-P3

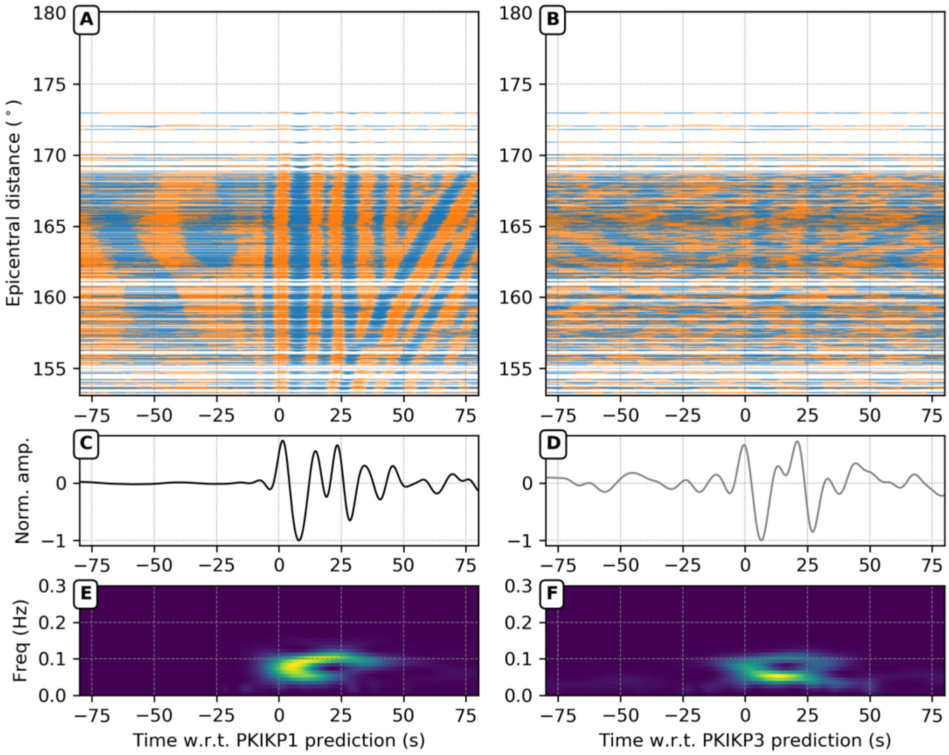

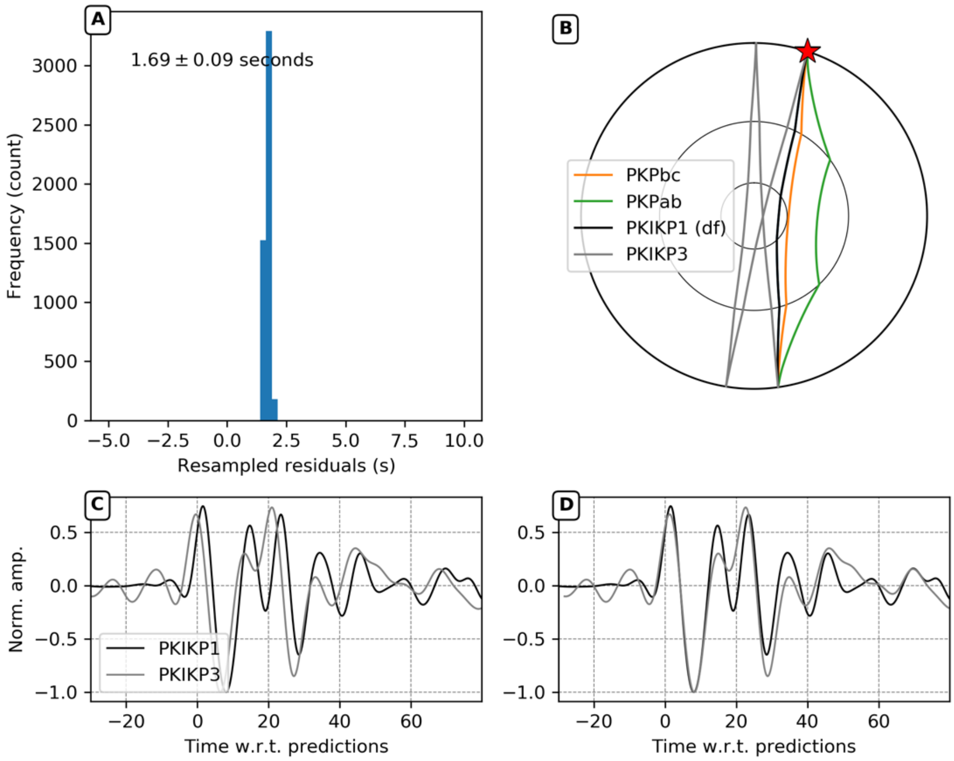

Event 20190619; Mantle correction model: DETOX-P3

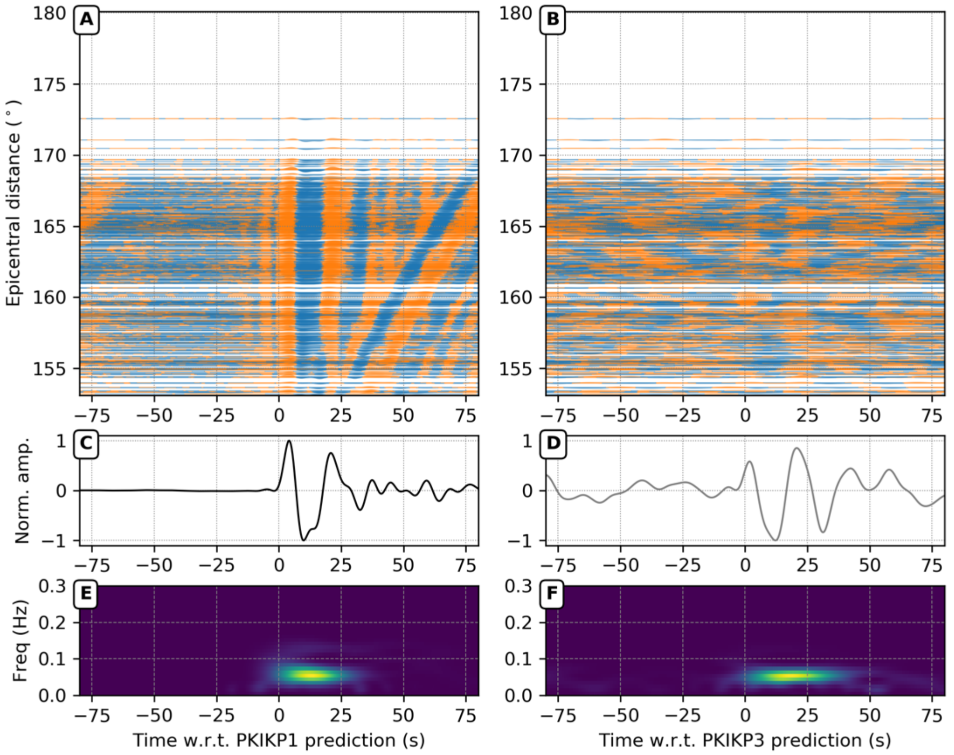

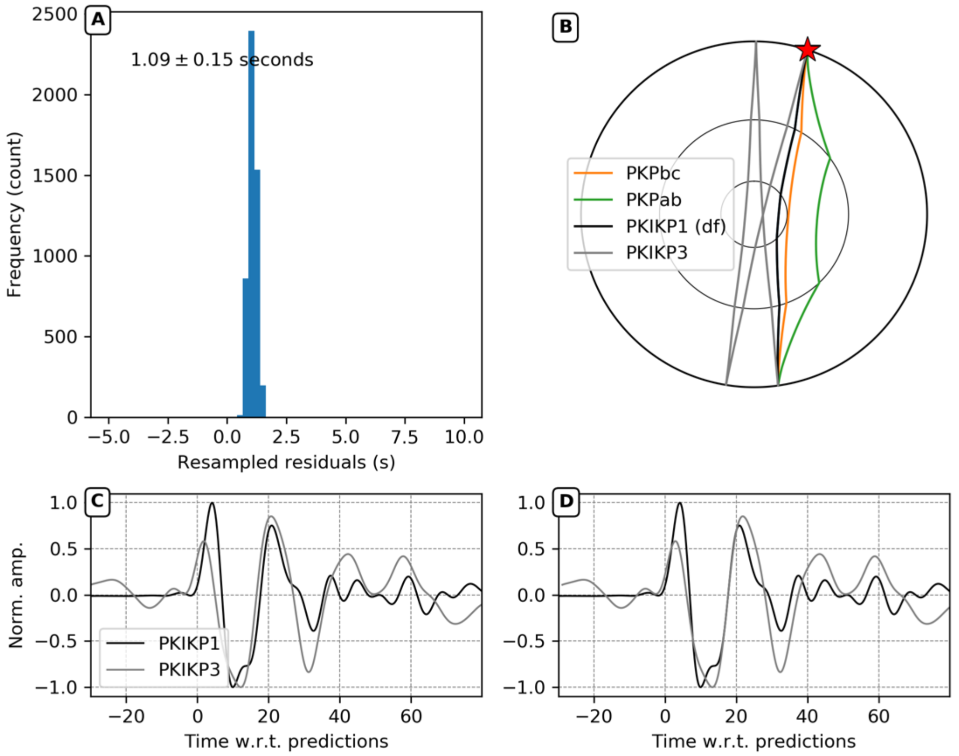

Supplement: Supplementary file 1 — Supporting Information [file 41467_2023_36074_MOESM1_ESM.pdf]
